# Assessing Older Adults' Adherence to Appropriate Polypharmacy: Selection of Outcome Measures for Intervention Trials

**DOI:** 10.1111/jgs.70313

**Published:** 2026-01-29

**Authors:** Hanadi A. Al Shaker, Heather E. Barry, Carmel M. Hughes

**Affiliations:** ^1^ Faculty of Pharmacy and Medical Sciences University of Petra Amman Jordan; ^2^ School of Pharmacy Queen's University Belfast Belfast UK

**Keywords:** adherence, core outcome set, interventions, older people, outcome measurement instruments, polypharmacy

## Abstract

**Background:**

Outcome measurement instruments (OMIs) are important for evaluating intervention effectiveness and quality. However, adopting OMIs remains challenging. This study aimed to select OMIs for a core outcome set (COS) for use in studies focusing on adherence to appropriate polypharmacy in older people.

**Methods:**

A list of OMIs for COS outcomes and their feasibility information was compiled from the literature to select one OMI per outcome. Two rounds of Delphi questionnaires containing a range of OMIs were distributed to experts [academics, healthcare professionals (HCPs), journal editors and methodologists] who were asked to select OMIs for a subsequent consensus meeting using ‘Yes’, ‘No’, or ‘Uncertain’. The Delphi results were discussed and OMIs were voted on (Yes: important and No: unimportant) in a consensus meeting with experts and an interview with a public member. An OMI was included if ≥ 80% of participants voted on it as critical and ≤ 20% voted it as unimportant.

**Results:**

Twenty‐one OMIs were presented to experts (Round 1, *n* = 42; Round 2, *n* = 39) in the Delphi exercise to achieve consensus on nine OMIs. Following the consensus meeting and interview (experts, *n* = 5; public participants, *n* = 1), agreement was achieved to select four OMIs: the Adherence to Refills and Medications Scale (ARMS, 100%); Multimorbidity Treatment Burden Questionnaire (MTBQ, 100%); Medication‐Related Burden Quality of Life questionnaire (MRB‐QoL, 83.3%); and ‘the number of undesired consequences of the intervention that result from administering multiple medications in older people (83.3%)’ for measuring medication adherence across multiple medications (subjective); treatment burden; health‐related quality of life (HRQoL) and adverse events and side effects (AEs and SEs), respectively. No agreement was reached regarding cost‐effectiveness and healthcare utilization.

**Conclusion:**

This study selected OMIs for use with a COS in studies to improve adherence to appropriate polypharmacy in older people. Future research should identify appropriate OMIs for the remaining outcomes.

## Summary

- Key points
  ○ Using consensus methodology, this study aimed to select outcome measurement instruments (OMIs) to be used in conjunction with a core outcome set for clinical trials aiming to improve adherence to appropriate polypharmacy.
  ○ Four OMIs were selected to assess medication adherence across multiple medications (subjective); treatment burden; health‐related quality of life; and adverse events and side effects.
  ○ Further research is required to identify suitable ways to measure other outcomes in the COS.
- Why does this paper matter?
  ○ This paper outlines the selection of measurement instruments for a COS for use in clinical trials aimed at improving adherence to appropriate polypharmacy in older people. Implementing this COS and its associated instruments should enhance transparency, harmonize research data, and facilitate evidence synthesis in systematic reviews in adherence to appropriate polypharmacy in older people.

## 1 Introduction

Medication non‐adherence has resulted in negative health outcomes in older patients (aged ≥ 65 years) taking polypharmacy (≥ 4 medications) [1]. Appropriate polypharmacy involves the administration of multiple medications that are prescribed according to the best available evidence [2]. Although existing literature outlines numerous methods to measure medication adherence, no single instrument has been deemed optimal due to inherent limitations [3]. A combination of subjective (e.g., self‐reported questionnaires) and objective measures (e.g., Electronic Monitoring Devices (EMDs)) has been recommended to deliver the most accurate adherence assessment [3]. Importantly, no universally accepted method has been used to measure adherence to polypharmacy [4]. Rather, the choice was influenced by data availability, disease type, researchers' preferences, and study setting [4]. Similarly, previous clinical trials aiming to improve adherence to polypharmacy in older people have shown significant heterogeneity in outcomes and outcome measurement instruments (OMIs) employed, leading to low‐quality evidence [1]. To address these inconsistencies, a core outcome set (COS) encompassing a standardized list of outcomes has been developed for trials seeking to improve adherence to appropriate polypharmacy in older people [5]; however, no research has specified ‘how to measure’ these outcomes.

An OMI is a Patient‐Reported Outcome Measure (PROM), a measurement method (e.g., number of hospitalisations) or a laboratory measure that evaluates health changes [6]. The COnsensus‐based Standards for the selection of health Measurement INstruments (COSMIN) initiative was established to guide researchers in selecting OMIs [6].

This study aimed to achieve consensus on OMIs associated with the COS for use in clinical trials seeking to improve adherence to appropriate polypharmacy in older people.

## 2 Methods

The scope of the COS covered intervention studies aiming to improve adherence to appropriate polypharmacy (≥ 4 medications) in community‐dwelling older people (aged ≥ 65 years) who live in their own homes (i.e., community‐dwelling patients) and who can manage their medications independently [5]. This study involved four phases:

### 2.1 Phase 1: Compilation of a List of OMIs Relevant to Adherence to Polypharmacy

Following the COSMIN guidelines recommendations, a list of OMIs was extracted from a Cochrane review targeting older people's adherence to polypharmacy [1] and another relevant study [7] for each outcome included in the COS: ‘medication adherence across multiple medications’, ‘all adverse events and side effects (AEs and SEs)’, ‘health‐related quality of life (HRQoL)’, ‘healthcare utilisation (HCU)’, and ‘cost‐effectiveness’ (Table S1). However, because treatment burden (TB) was not assessed in the previous studies related to adherence to polypharmacy [1], the COSMIN database for systematic reviews (https://database.cosmin.nl/) was consulted to identify all generic TB‐related OMIs using the search term (treatment burden) and selecting the senior's category from 2005 to 2024. We also aimed to include one objective and one subjective adherence measure to obtain an accurate adherence assessment.

Given the existence of multiple chronic conditions that lead to polypharmacy, the psychometric properties of PROMs included in this study were not assessed.

### 2.2 Phase 2: Identifying Feasibility Aspects and Information Card Preparation

‘Feasibility’ is the practicality and ease of implementing an instrument in clinical settings while considering constraints (e.g., length of administration and cost) [8]. Following a comprehensive literature search [9, 10, 11], information cards were prepared to ensure transparency and provide summaries of each OMI, including feasibility details (e.g., recall period, completion time, and administration mode). The layout and structure were informed by information cards prepared for two other studies [9, 10].

### 2.3 Phase 3: The Delphi Exercise

A two‐round sequential Delphi study was performed to select the two highest‐scoring ‘consensus in’ OMIs per outcome. Experts were identified from publication records and journal editorial board information and included: (1) academics with knowledge in polypharmacy, adherence, older people and OMIs; (2) HCPs, namely pharmacists, doctors, and nurses who deliver care to older people; and (3) methodologists who developed PROMs and/or conducted a previous COSMIN review that evaluated the quality of PROMs considered in this study; (4) editors of peer‐reviewed journals targeting adherence, polypharmacy, and gerontology (Table S2). Snowball sampling was also employed to enhance recruitment. There is no guidance for the number of participants in a Delphi panel, and previous studies have ranged from 35 to 213 [12, 13]. Furthermore, because only 53 participants took part in our previous Delphi study [5], we anticipated that around 35 to 65 experts would participate in Round 1 of this present study. The Delphi questionnaires were piloted with five researchers at Queen's University Belfast (QUB) and refined based on feedback.

Following distribution of invitation emails and study information sheets, those who agreed to participate received a first‐round email containing their identification numbers and an online questionnaire link circulated through the SoGolytics platform. Experts completed consent and demographic details forms. The questionnaire included an overview of the study and completion instructions, followed by the list of the COS's outcomes, their definitions, and all identified OMIs, along with their information cards.

Additionally, experts were invited to suggest other OMIs for each outcome. It was agreed to include OMIs deemed generic instruments, not already provided by the team and recommended by four or more experts. After each round, the response rate and score distribution for each OMI were calculated. The Delphi questionnaire adopted three voting categories (‘yes’, ‘no’, and ‘uncertain’). A text box appeared if a participant chose ‘no’ or ‘uncertain’ to justify their selection. OMIs voted on as ‘yes’ by ≥ 80% of the participants and ‘no’ by ≤ 20% of the participants were included in the final list of OMIs and were classified as ‘consensus in’, whereas OMIs voted on as ‘no’ by ≥ 80% of the participants and ‘yes’ by ≤ 20% of the participants were excluded from the final list of OMIs and were classified as ‘consensus out’. OMIs that did not achieve either ‘consensus in’ or ‘consensus out’ were classified as ‘no consensus’. Round 2 included all ‘consensus in’ and ‘no consensus’ OMIs from Round 1, any suggested OMIs approved by the research team, personalized feedback reports, and a group feedback report to enable comparisons. After Round 2, only OMIs meeting the inclusion threshold were included and considered in the consensus meeting.

### 2.4 Phase 4: The Consensus Meetings

We planned to conduct two separate consensus meetings for experts and public participants (PPs) to agree on one OMI for each outcome. PPs were recruited from older people's foundations, associations, organizations, and charities, as well as staff of older patients' organizations advocating for older patients' interest in healthcare and who had publicly available contact information (e.g., email addresses). Information cards for PPs were prepared for OMIs that achieved ‘consensus in’ thresholds from Phase 3. Sampling and recruitment of experts were performed as in the Delphi exercise. PPs were approached by searching for publicly available email addresses of staff working in organizations concerned with supporting older people (Table S2). These organizations were also asked to assist with PP recruitment.

Due to the lack of guidance on optimal participant numbers for consensus meetings and based on our previous research [5], we anticipated 10–15 participants taking part in both meetings. The consensus process, workbooks and voting questionnaires were piloted with six researchers at QUB and Queen Mary University of London. Events were then hosted through Microsoft Teams using the SoGolytics platform to facilitate data collection.

Ethical approval was granted for the Delphi exercise and consensus meetings by the Faculty of Medicine, Health and Life Sciences Research Ethics Committee, QUB (reference number: MHLS 24_123).

#### 2.4.1 The Consensus Meeting Process

**FIGURE 1:**
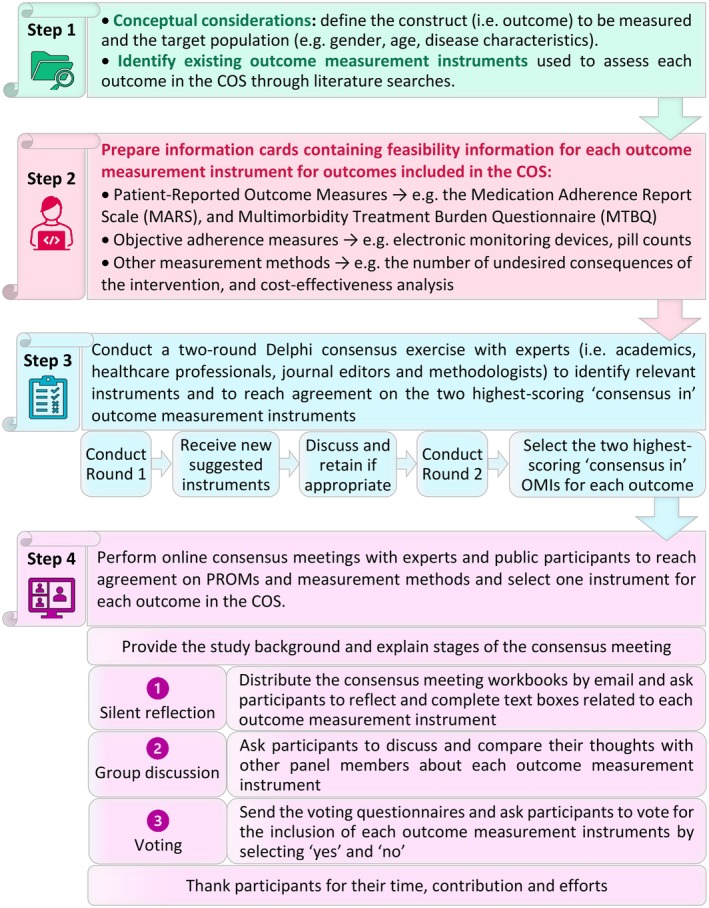
The main steps involved in the selection of outcome measurement instruments (OMIs) for a Core Outcome Set (COS) for clinical trials targeting interventions aiming to improve adherence to appropriate polypharmacy in older people. In *Step 1*, the construct (e.g., medication adherence across multiple medications, cost‐effectiveness, health‐related quality of life) and the target population (e.g., older people) were determined. OMIs used to measure each outcome in the COS were also identified. *Step 2* involved the preparation of all information cards for each OMIs for the COS's outcomes. In *Step 3*, a two‐round Delphi consensus exercise was undertaken with experts to achieve agreement on the two highest‐scoring ‘consensus in’ OMIs. *Step 4* involved online consensus meetings with experts and public members to select one instrument for each outcome in the COS.

Two scripts were prepared for each participant group (Supporting Information S1), following a four‐stage process: introduction, silent reflection, group discussion, and voting (Figure 1). After the introduction, participants received identification numbers and a link to workbooks presenting the outcomes, definitions, the Delphi study ‘consensus in’ OMIs, information cards and a text box to document their silent reflection responses on the OMIs. Following submission of workbooks via the online platform, participants received individualized reports presenting their responses. Lastly, participants received questionnaires to vote on each OMI anonymously by selecting ‘yes’ and ‘no’ for inclusion. Consensus was reached when an OMI was voted on as ‘yes’ by ≥ 80% of panelists and ‘no’ by ≤ 20% of panelists. The consensus meeting finalized the list of OMIs, which only included ‘consensus in’ OMIs. The process of selecting OMIs is summarized in Figure 1.

## 3 Results

### 3.1 Phase 1: Compilation of a List of OMIs Relevant to Adherence to Polypharmacy

A list of 13 PROMs, five objective adherence measures and three measurement methods were compiled from a previous study and systematic reviews [1, 7, 14, 15] (Table 1).

**TABLE 1:** The final list of outcomes included in the COS, along with the identified outcome measurement instruments compiled from systematic reviews [1, 14, 15] and another relevant study [7].

Outcomes	Outcome measurement instruments
Medication adherence across multiple medications	PROMs:
	1. BMQ
	2. MARS
	3. The MOS—Specific Adherence Scale
	4. MMAS‐4
	Objective adherence measures:
	5. EMDs
	6. Pill counts
	7. MPR
	8. PDC
	9. DPPR
Treatment burden	PROMs:
	1. TBQ
	2. MTBQ
	3. PETS
	4. LMQ‐3
Health‐related quality of life	PROMs:
	1. EQ‐5D‐3L
	2. EQ‐5D‐5L
	3. SF‐12
	4. SF‐36
	5. MRB‐QoL
All adverse events and side effects	Measurement method:
	The number of undesired consequences of the intervention (i.e., adverse events or side effects) that result from administering multiple medications in older patients
Healthcare utilization	Measurement method:
	The number or percentage of a specified utilized service during/in a specified time period that results from administering multiple medications in older patients
Cost‐effectiveness	Measurement method:
	Consulting a health economist about the most appropriate method

Abbreviations: BMQ, The Brief Medication Questionnaire; DPPR, Daily Polypharmacy Possession Ratio; EMDs, Electronic Monitoring Devices; EQ‐5D‐3L, The 3‐level EQ‐5D questionnaire; EQ‐5D‐5L, The 5‐level EQ‐5D questionnaire; LMQ‐3, The Living with Medicines Questionnaire‐3; MARS, Medication Adherence Report Scale; MMAS‐4, The Morisky Medication Adherence Scale‐4; MOS, The Medical Outcome Study; MPR, The Medication Possession Ratio; MRB‐QoL, The Medication‐Related Burden Quality of Life questionnaire; MTBQ, Multimorbidity Treatment Burden Questionnaire; PDC, The Proportion of Days Covered; PROMs, Patient‐Reported Outcome Measures; PETS, Patient Experience with Treatment and Self‐management Questionnaire; SF‐12, The Short Form‐12; SF‐36, The Short Form‐36; TBQ, Treatment Burden Questionnaire.

### 3.2 Phase 2: Identifying Feasibility Aspects and Information Card Preparation

Feasibility aspects were compiled for all identified OMIs, and experts' information cards were prepared for presentation in the Delphi study (https://adherence2polypharmacy‐instruments.org/).

### 3.3 Phase 3: The Delphi Exercise

Of the 320 experts invited to participate, only 43 accepted the invitation, with two additional experts recruited via snowball sampling. Of those 45 respondents, 42 (response rate = 93.3%) experts completed the first‐round questionnaire. Table 2 outlines participants' demographic details for the Delphi exercise.

**TABLE 2:** Demographic details of participants in the Delphi study and the consensus meeting/interview.

Participant characteristics	Delphi consensus exercise		The consensus meeting/interview
	Round 1	Round 2	
Participant, *n*	42	39	6
Age in years, median (range)	39 (27–73)	38 (27–73)	56 (33–73)
Gender, *n* (%)			
Men	13 (31.0)	12 (30.8)	2 (33.3)
Women	29 (69.0)	27 (69.2)	4 (66.6)
Continent of residence, *n* (%)			
America	6 (14.3)	6 (15.4)	0 (0.0)
Asia	7 (16.7)	6 (15.4)	0 (0.0)
Australia	6 (14.3)	5 (12.8)	0 (0.0)
Europe	23 (54.8)	22 (56.4)	6 (100.0)
Professional area, *n* (%)			
Doctor	3 (7.1)	2 (5.1)	1 (16.7)
Pharmacist	16 (38.1)	16 (41.0)	1 (16.7)
Academic	21 (50.0)	19 (48.7)	2 (33.3)
Pharmacist and academic	2 (4.8)	2 (5.1)	1 (16.7)
Public participant	—	—	1 (16.7)

Following Round 1, only one method reached consensus for inclusion, while five PROMs were excluded (Table 3). Respondents provided different reasons for selecting ‘no’ and ‘uncertain’ (Table S3) and suggested several OMIs for each outcome (Table S4).

**TABLE 3:** Degree of importance for each outcome measurement instrument following the Delphi rounds and the consensus meeting/interview.

The Delphi consensus exercise							The consensus meeting/interview		
Outcomes and OMIs	Round 1			Round 2			Voting by 6 participants, *n* (%)		
	Rating by 42 participants, *n* (%)			Rating by 39 participants, *n* (%)					
	Yes	No	Uncertain	Yes	No	Uncertain	Yes	No	Consensus results (80%/20%)^a^
Medication adherence across multiple medications									
**Subjective adherence measures (PROMs)**									
1. BMQ	3 (7.1)	34 (81.0)	5 (11.9)	✗	✗	✗	✗	✗	✗
2. MARS	26 (61.9)	5 (11.9)	11 (26.2)	27 (69.2)	8 (20.5)	4 (10.3)	✗	✗	✗
3. The MOS Scale	4 (9.5)	34 (81.0)	4 (9.5)	✗	✗	✗	✗	✗	✗
4. MMAS‐4	5 (11.9)	34 (81.0)	3 (7.1)	✗	✗	✗	✗	✗	✗
**Objective adherence measures**									
5. EMDs	14 (33.3)	19 (45.2)	9 (21.4)	3 (7.7)	29 (74.4)	7 (17.9)	✗	✗	✗
6. Pill counts	18 (42.9)	16 (38.1)	8 (19.0)	13 (33.3)	18 (46.2)	8 (20.5)	✗	✗	✗
7. MPR	21 (50.0)	11 (26.2)	10 (23.8)	23 (59.0)	12 (30.8)	4 (10.3)	✗	✗	✗
8. PDC	23 (54.8)	6 (14.3)	13 (31.0)	26 (66.7)	7 (17.9)	6 (15.4)	✗	✗	✗
9. DPPR	22 (52.4)	5 (11.9)	15 (35.7)	29 (74.4)	4 (10.3)	6 (15.4)	2 (33.3)	4 (66.7)	No consensus
**Suggested PROMs (added to Round 2)**									
10. ARMS	—	—	—	32 (82.1)^b^	2 (5.1)	5 (12.8)	6 (100.0)^d^	0 (0.0)	Consensus in
11. MAUQ	—	—	—	9 (23.1)	19 (48.7)	11 (28.2)	✗	✗	✗
12. MAR‐Scale	—	—	—	20 (51.3)	7 (17.9)	12 (30.8)	✗	✗	✗
**Treatment burden**									
1. TBQ	5 (11.9)	34 (81.0)	3 (7.1)	✗	✗	✗	✗	✗	✗
2. MTBQ	32 (76.2)	5 (11.9)	5 (11.9)	37 (94.9)^b^	1 (2.6)	1 (2.6)	6 (100.0)^d^	0 (0.0)	Consensus in
3. PETS	5 (11.9)	34 (81.0)	3 (7.1)	✗	✗	✗	✗	✗	✗
4. LMQ‐3	20 (47.6)	8 (19.0)	14 (33.3)	32 (82.1)^b^	3 (7.7)	4 (10.3)	2 (33.3)	4 (66.7)	No consensus
**Health‐related quality of life**									
1. EQ‐5D‐3L	16 (38.1)	22 (52.4)	4 (9.5)	6 (15.4)	30 (76.9)	3 (7.7)	✗	✗	✗
2. EQ‐5D‐5L	31 (73.8)	4 (9.5)	7 (16.7)	32 (82.1)^b^	4 (10.3)	3 (7.7)	4 (66.7)	2 (33.3)	No consensus
3. SF‐12	15 (35.7)	19 (45.2)	8 (19.0)	8 (20.5)	26 (66.7)	5 (12.8)	✗	✗	✗
4. SF‐36	15 (35.7)	16 (38.1)	11 (26.2)	5 (12.8)	28 (71.8)	6 (15.4)	✗	✗	✗
5. MRB‐QoL	23 (54.8)	10 (23.8)	9 (21.4)	32 (82.1)^b^	5 (12.8)	2 (5.1)	5 (83.3)^d^	1 (16.7)	Consensus in
**All adverse events and side effects**: The number of undesired consequences of the intervention (i.e., adverse events or side effects) that result from administering multiple medications in older patients									
—	36 (85.7)^c^	1 (2.4)	5 (11.9)	39 (100.0)^c^	0 (0.0)	0 (0.0)	5 (83.3)^d^	1 (16.7)	Consensus in
**Healthcare utilization**: The number or percentage of a specified utilized service during/in a specified time period that results from administering multiple medications in older patients									
—	28 (66.7)	4 (9.5)	10 (23.8)	37 (94.9)^b^	1 (2.6)	2 (2.6)	3 (50.0)	3 (50.0)	No consensus
**Cost‐effectiveness**: Consulting a health economist about the most appropriate method									
—	33 (78.6)	3 (7.1)	6 (14.3)	36 (92.3)^b^	3 (7.7)	0 (0.0)	2 (33.3)	4 (66.7)	No consensus

*Note*: ✗ Outcome measurement instruments that were not carried forward from Round 1 to 2 or from Round 2 to the consensus meeting.

Abbreviations: ARMS, The Adherence to Refills and Medications Scale; BMQ, The Brief Medication Questionnaire; DPPR, Daily Polypharmacy Possession Ratio; EMDs, Electronic Monitoring Devices; EQ‐5D‐3L, The 3‐level EQ‐5D questionnaire; EQ‐5D‐5L, The 5‐level EQ‐5D questionnaire; LMQ‐3, The Living with Medicines Questionnaire‐3; MARS, Medication Adherence Report Scale; MMAS‐4, The Morisky Medication Adherence Scale‐4; MOS, The Medical Outcome Study—Specific Adherence Scale; MPR, The Medication Possession Ratio; MRB‐QoL, The Medication‐Related Burden Quality of Life questionnaire; MTBQ, Multimorbidity Treatment Burden Questionnaire; OMIs, outcome measurement instruments; PDC, The Proportion of Days Covered; PETS, Patient Experience with Treatment and Self‐management Questionnaire; PROMs, Patient‐Reported Outcome Measures; SF‐12, The Short Form‐12; SF‐36, The Short Form‐36; TBQ, Treatment Burden Questionnaire.

^a^ Outcome measurement instruments voted on as ‘yes’ by ≥ 80% of the participants AND ‘no’ by ≤ 20% of the participants were included in the final list of outcome measurement instruments, whereas outcome measurement instruments voted on as ‘no’ by ≥ 80% of the participants AND ‘yes’ by ≤ 20% of the participants were excluded from the final of outcome measurement instruments. Outcome measurement instruments which achieved ‘consensus in’ and ‘no consensus’ were carried over from Rounds 1 to 2, while only ‘consensus in’ OMIs were carried over from Round 2 to the consensus meeting. The consensus meeting included ‘consensus in’ OMIs.

^b^ Figures representing the measurement instruments that achieved the threshold for inclusion after Round 2.

^c^ Figures representing the measurement instruments that achieved the threshold for inclusion after Rounds 1 and 2.

^d^ Figures representing the measurement instruments that achieved the threshold for inclusion after the consensus meeting and were therefore included in the final list of outcome measurement instruments.

All ‘consensus in’ and ‘no consensus’ OMIs presented in Round 1, along with three suggested adherence‐related PROMs approved by the research team, progressed to Round 2. In Round 2 (*n* = 39; response rate = 92.9%), five PROMs, including the Adherence to Refills and Medications Scale (ARMS), Multimorbidity Treatment Burden Questionnaire (MTBQ), the Living with Medicines Questionnaire‐3 (LMQ‐3), EuroQoL 5‐Dimension 5‐Level (EQ‐5D‐5L), and Medication‐Related Burden Quality of Life (MRB‐QoL), and three measurement methods related to AEs and SEs, HCU, and cost‐effectiveness reached consensus for inclusion in the list of OMIs. However, consensus on objective adherence measures was not reached. Therefore, the research team endorsed the inclusion of the Daily Polypharmacy Possession Ratio (DPPR), given the scope of adherence to appropriate polypharmacy COS. Table 3 outlines the distribution of scores for each OMI for the Delphi consensus exercise.

### 3.4 Phase 4: The Consensus Meetings

Of the 300 experts and 25 PPs who received the invitations, 14 experts agreed to participate, with no responses from PPs. Additionally, 1261 members of the British Geriatrics Society received invitations via snowball sampling, whereby an additional 14 individuals accepted the invitations. However, because of the limited availability of experts, only five were able to join the online consensus meeting. Of the 42 PP organizations contacted, only one agreed to distribute the invitations to their members, resulting in one individual consenting to participate. Therefore, the meeting followed an interview‐like format. Both the expert and PP sessions lasted approximately 1 h and 20 min.

#### 3.4.1 The Workbooks

Participants reported their responses regarding each OMI as outlined in Table 4, which displays all OMIs for each outcome, along with their clarifications and excerpts of the responses. Table S5 also shows the silent reflection responses as reported in the SoGolytics platform.

**TABLE 4:** Participants' silent reflection responses about all outcome measurement instruments, along with brief commentary on each excerpt.

OMIs	Participants' excerpt/s and explanation
**1. Medication adherence across multiple medications**	
Subjective adherence measure: ARMS	Participants believed that the ARMS would be a reliable measure to assess medication non‐adherence as it was deemed comprehensive. “*I agree that the ARMS can provide reliable data*” [P2] “*Yes. I like ARMS. Good for low literacy. Covers a range of points (thorough)*.” [P4] However, it was noted that the ARMS failed to examine medication non‐adherence‐related reasons. “*The questionaire [*sic*] does not explore why patients are missing their medication*” [P5]
Objective adherence measures: DPPR	Although some participants reported that the DPPR could be used to measure non‐adherence, others raised concerns regarding its accuracy since ordering medications does not guarantee that the patient had taken them. “*no confirmation on medication taking, what's collected from the chemist could or [*sic*] sold or given to another*.” [PP1]
**2. Treatment burden**	
MTBQ and the LMQ‐3	The majority of participants believed that the MTBQ was better for measuring treatment burden than the LMQ‐3 due to its conciseness, validation and availability in multiple languages. “*I would advice [*sic*] MTBQ for two reasons: 1. it is short 2. availability of validated versions in multiple languages*” [P2]
**3. Health‐related quality of life**	
EQ‐5D‐5L and the MRB‐Qol	There were conflicting responses regarding the MRB‐QoL and EQ‐5D‐5L. Some participants preferred using the MRB‐QoL due to its polypharmacy‐specific questions and comprehensiveness, which would facilitate the collection of more detailed medication‐related information. However, it was deemed lengthy; therefore, others reported that the EQ‐5D‐5L was more suitable for measuring health‐related quality of life. Participants also stated that the EQ‐5D‐5L was concise and available in multiple languages, facilitating comparisons between studies, and could be used to estimate cost‐effectiveness. “*The MRB‐QoL is better for research to know simple differences in adherence to polypharmacy*.” [P1] “*For reasons of comparability to previous studies, I would advice [*sic*] to use EQ‐5D‐5L”* [P2] “*My preference is the EQ‐5D‐%L [*sic*] because it is short, robust, and translated*” [P3]
**4. All adverse events and side effects**	
The number of undesired consequences of the intervention (i.e., adverse events or side effects) that result from administering multiple medications in older patients	Many participants noted that ‘the number of undesired consequences of the intervention’ could be used to measure all adverse events and side effects. “*This is [*sic*] obligation for healthcare workers. We must look for them also to improve our advices [*sic*] to patient (I was doing it through my 40 years practice!)*.” [P1] However, others deemed measuring this outcome challenging, given the difficulty of connecting these side effects and adverse events with a specific underlying reason (e.g., disease or drug). “*Would not use this because it is to [*sic*] difficult [*sic*] for patients to connect side‐effects to drugs most of the time. And it is not very specific”* [P3]
**5. Healthcare utilization**	
The number or percentage of a specified utilized service during/in a specified time period that results from administering multiple medications in older patients	Participants highlighted the need to specify the type of healthcare services that should be used in trials to measure healthcare utilization. “*No, there is not always a clear relation between adherence and healthcare utilization and utilization is often very difficult do [*sic*] assess and it needs a clear and precise clarification of health care services types*.” [P3]
**6. Cost‐effectiveness**	
Consulting a health economist about the most appropriate method	The majority of participants lacked the necessary knowledge and background regarding cost‐effectiveness and how it was evaluated. “*Not familiar with this, unable to comment*.” [P5]

Abbreviations: ARMS, The Adherence to Refills and Medications Scale; DPPR, Daily Polypharmacy Possession Ratio; EQ‐5D‐5L, The 5‐level EQ‐5D questionnaire; LMQ‐3, The Living with Medicines Questionnaire‐3; MRB‐QoL, The Medication‐Related Burden Quality of Life questionnaire; MTBQ, Multimorbidity Treatment Burden Questionnaire.

#### 3.4.2 The Voting Questionnaires

All six participants (five experts and one PP) completed the voting questionnaires during their respective sessions. Three PROMs (ARMS, MTBQ, MRB‐QoL) and one measurement method [the number of undesired consequences of the intervention (i.e., AEs and SEs) that result from administering multiple medications in older people] met the threshold for inclusion to measure medication adherence across multiple medications, TB, HRQoL, and AEs and SEs, respectively. The remaining OMIs did not reach consensus and were therefore excluded (Table 3).

Supporting Information Figure S1 displays a comprehensive flow chart summarizing the identification and selection of OMIs for a COS for clinical trials targeting interventions to improve adherence to appropriate polypharmacy in older people.

## 4 Discussion

This international consensus‐based study has selected OMIs for each outcome included in the COS for use in clinical trials to enhance adherence to appropriate polypharmacy in older people. The resultant nine OMIs that achieved consensus after the second Delphi round progressed to the consensus meetings, namely the ARMS, LMQ‐3, MTBQ, EQ‐5D‐5L and MRB‐QoL, and measurement methods related to AEs and SEs, HCU and cost‐effectiveness (Table 3). Although the DPPR did not achieve consensus, its unique ability to measure adherence in patients taking polypharmacy [16] and the relatively high consensus score (74.4% voted ‘yes’ for inclusion in Round 2) led to its consideration in the consensus meeting. However, following the final meeting, only the ARMS, MTBQ, MRB‐QoL, and ‘the number of undesired consequences of the intervention (i.e., AEs or SEs) that result from administering multiple medications in older people’ were included in the final list to assess medication adherence across multiple medications, TB, HRQoL and AEs and SEs, respectively.

Instrument selection for this work followed a systematic approach by identifying existing OMIs from previous research, determining feasibility aspects and preparing information cards. Tegegn and colleagues revealed that the ARMS or ARMS‐7 had high‐quality evidence for structural and construct validity, reliability, and internal consistency, making both PROMs the most suitable for patients with cardiovascular diseases [17]. Surprisingly, participants suggested the ARMS after the first round and voted for its inclusion. This highlighted their long‐standing knowledge and familiarity with the available PROMs gained through research experience. The ARMS and ARMS‐7 also provide comprehensive adherence assessment by exploring the reasons (intentional or unintentional) and the extent of non‐adherence (whether the prescriptions are filled or taken as prescribed) [17]. This questionnaire evaluates challenges to appropriate administration, forgetfulness, medication‐taking behavior, and prescription refill ability [18, 19]. Nevertheless, as highlighted in the meeting, it does not identify all other adherence‐related barriers (e.g., social support) [18]. The ARMS, like many other adherence questionnaires, assesses adherence during the implementation phase and the discontinuation phase, but not the initiation phase [17]. This pattern was clearly reflected in Tegegn et al., where all PROMs were shown to capture patients' self‐reported behaviors primarily during the implementation phase, but not the initiation or persistence phases [17].

Although we sought to include one subjective and objective measure for adherence, none of the presented objective measures achieved consensus in this study. The DPPR was deemed inaccurate as it failed to ensure medication ingestion. Nevertheless, retrospective databases can evaluate medication initiation and discontinuation [20, 21], which is an advantage over questionnaires that cannot measure initiation [17].

Despite receiving the lowest consensus level in the Delphi exercise, EMDs have been widely acknowledged as a standard measure for assessing adherence when combined with retrospective databases [20]. Selecting one adherence measure would be an unfeasible task, given the limitations associated with each measure. Measuring adherence should involve a combination of subjective and objective measures while considering adherence behavior timelines (i.e., initiation, implementation, and discontinuation) and challenges [20, 22].

A COSMIN review of TB‐related PROMs used in multimorbidity revealed there was little supporting evidence; therefore, no single PROM was recommended for use in TB [14]. Furthermore, all the available TB‐related PROMs, such as the MTBQ, overlooked some TB‐related components for people with multimorbidity, for example, emotional burden [23, 24]. However, participants deemed the MTBQ an appropriate PROM because it was explicitly developed for multimorbidity, translated and validated in multiple languages, comprised 10 items and had a simple scoring system (https://adherence2polypharmacy‐instruments.org/). The MTBQ demonstrated sufficient content and construct validity, reliability and responsiveness, enabling it to detect pre‐ and post‐intervention changes [14].

Additionally, the MRB‐QoL was deemed the only HRQoL questionnaire targeting polypharmacy‐related challenges and burdens. However, it was considered lengthy; thus, some participants preferred the EQ‐5D‐5L due to its brevity, multilingual versions, and its use in cost‐effectiveness analyses. Combining certain items from each questionnaire is preferred to comprehensively measure the effectiveness of interventions on adherence to appropriate polypharmacy trials. This was performed in a previous feasibility study aimed at assessing the effect of the PolyPrime intervention to improve appropriate polypharmacy for older adults, where specific items from each questionnaire were used to evaluate HRQoL [25]. However, the 31 items of MRB‐QoL led to more missing data than were observed in the EQ‐5D‐5L [25]. Combining certain items from both questionnaires is recommended to comprehensively measure the effectiveness of interventions on adherence to appropriate polypharmacy trials.

Of the measurement methods used to assess AEs and SEs, only ‘the number of undesired consequences that result from administering multiple medications in older people’ received support for inclusion in the Delphi questionnaires and the consensus meeting. However, identifying the root cause of AEs in the older population would be challenging due to various pharmacokinetic, physical and cognitive‐related changes [26]. Thus, instead of recommending one definite OMI, a trial‐specific method could be adopted to align with the study's aim, settings and design.

Participants considered the presented HCU‐related measure vague, as it failed to define the type of services utilized. Thus, it was excluded following the consensus meeting. Indeed, multiple HCU‐related questionnaires have been developed and employed for specific diseases, target populations, or certain utilized services [27]. However, none of these PROMs have been used to measure HCU in the context of adherence to polypharmacy in older patients [1]. Leggett and colleagues also highlighted the importance of combining administrative data (e.g., hospital admission records) with questionnaires as a benchmark to ensure accurate assessment [27]. Accordingly, we would advise combining self‐reported questionnaires and administrative data tailored to reflect the study's design, aim, and healthcare services utilized.

‘Consulting a health economist about the most appropriate method’ was excluded after the consensus meeting; however, this was attributed to the participants' limited knowledge and unfamiliarity with the approach rather than a methodological concern. Another possible reason is that we did not use a specific economic evaluation measure to define cost‐effectiveness in the information card. Instead, cost‐effectiveness analysis was defined as a broad framework that draws on data from resource use and HRQoL, with the quality‐adjusted life year (QALY) identified as one potential measure that is not universal. Earlier trials addressing adherence to polypharmacy in older people did not adopt a single international approach [1]. Similarly, a systematic review examining medication non‐adherence across disease groups demonstrated that methodological inconsistencies, such as differences in adherence measurement instruments, cost‐reporting methods, and economic evaluation quality, impeded comparability and the accurate estimation of non‐adherence‐related costs [28]. Therefore, we emphasized that the calculation and interpretation of cost‐effectiveness require trained researchers and ideally consultation with a health economist to ensure methodological rigor and contextual appropriateness. This approach was also agreed upon in a study that evaluated the effectiveness of fall prevention interventions in people with Parkinson's disease, multiple sclerosis, and stroke [29].

This work has several strengths. First, this study differs from other consensus‐based studies in that it targeted adherence to appropriate polypharmacy in older people rather than medication adherence to a single disease or drug in adults. Older people are a population whose medication‐taking behavior and barriers differ from the general adult population due to polypharmacy that introduces additional difficulties to this population (e.g., drug interactions, multiple doses) as well as the influence of cognitive, social, and physical challenges [1]. Another strength is the diverse representation of the Delphi panel from 17 countries based in Asia, America, Europe, and Australia, which would improve the generalisability of findings worldwide. The panel comprised academics with expertise in research on geriatric medication management, COSMIN review authors, and HCPs from two professional experiences in healthcare (e.g., pharmacists and doctors). Another strength was the selected consensus definition (80%/20%), which ensured the inclusion of OMIs deemed the most feasible and appropriate. Finally, the high response rate for both rounds (93.3% and 92.9%, respectively) facilitated thorough data collection and decreased sample bias [30].

However, this study has a number of key limitations. First, despite approaching a large pool of individuals for the Delphi exercise and the consensus meeting (*n* = 320 and *n* = 300, respectively), limited numbers of experts participated (*n* = 42 and *n* = 5, respectively). Similarly, only one PP took part in the consensus meeting despite circulating invitations via snowball sampling to 1261 British Geriatrics Society members, and approaching 42 public involvement organizations. The meeting's expected duration (over an hour) and the PP institutions' focus on other priorities rather than research may explain the lack of response. To enhance future public participation, researchers might consider older people's and carers' Facebook groups that target chronic conditions (e.g., Heart Failure Support Group). Second, the Delphi questionnaires and the consensus meeting, along with their information cards, were conducted in English, and the contacted older people's charities/organizations were also located in English‐speaking countries. This approach had been taken for pragmatic reasons, which may have reduced the number of PPs. Third, we did not conduct a formal subgroup analysis to determine whether participants preferred specific instruments over others, given the small sample size. Therefore, we focused on overall group consensus rather than analyzing professional‐specific preferences. Finally, a formal risk of bias assessment of the measurement properties of PROMs was not conducted. This decision was informed by the available validation evidence, whereby searches of the COSMIN database for systematic reviews evaluating adherence‐related PROMs, HRQoL instruments, and TB measures revealed significant heterogeneity in the quality of measurement properties across various chronic conditions [14, 15, 17, 31, 32, 33, 34]. The validity of the majority of available PROMs was rated as poor, leading some systematic reviewers to recommend PROMs with “potential for use” rather than instruments with well‐established, high‐quality measurement properties [33]. Importantly, instead of evaluating the existing PROMs retrospectively, future research should prioritize the development of new PROMs with robust validation methodologies tailored to the polypharmacy context.

A list of OMIs has been developed for trials seeking to improve adherence to appropriate polypharmacy in older people. The OMIs ‘ARMS’, ‘MTBQ’, ‘MRB‐QoL’ and ‘number of undesired consequences of the intervention that result from administering multiple medications in older people’ were selected for ‘medication adherence across multiple medications’, ‘TB’, ‘HRQoL’, and ‘AEs and SEs’, respectively. It is recommended that this list be used in future trials to enhance the consistency and quality of findings and minimize OMIs‐reporting bias. Future work is needed to select OMIs for cost‐effectiveness, HCU, and objective adherence measures.

## Author Contributions

Carmel M. Hughes conceived the idea for the study. All authors devised the study protocol. Hanadi A. Al Shaker was responsible for running the two rounds of the Delphi questionnaires and consensus meetings. All authors contributed to project management. Hanadi A. Al Shaker conducted the literature review, identified the outcome measurement instruments from previous research, and prepared the information cards for the Delphi questionnaires and consensus meetings. All authors developed the Delphi questionnaires and the content of the consensus meeting workbook and voting questionnaires. Data analysis was undertaken by Hanadi A. Al Shaker and checked by Carmel M. Hughes and Heather E. Barry. Hanadi A. Al Shaker drafted the manuscript. All authors revised and reviewed the manuscript. Data for this work is accessible to all authors. All authors are responsible for submitting it for publication.

## Funding

This work was supported by the University of Petra.

## Ethics Statement

Ethical approval was granted by the Queen's University Belfast Ethics Committee of the Faculty of Medicine, Health and Life Sciences, QUB (reference number: MHLS 24_123).

## Consent

All participants consented before participating in the Delphi exercise and the consensus meetings. Participants in the consensus meetings were aware that workbook quotations may be used; however, all information has been anonymized.

## Conflicts of Interest

The authors declare no conflicts of interest.

## Supporting information


**Data S1:** Supporting Information.

## Data Availability

Anonymized datasets obtained during and/or analyzed during this study are available from the corresponding author upon reasonable request.
