# Supplementary material for: Assessing Older Adults' Adherence to Appropriate Polypharmacy: Selection of Outcome Measures for Intervention Trials
Source: J Am Geriatr Soc. 2026 Jan 29;74(3):781–92. doi: 10.1111/jgs.70313 (PMC12968370; doi:10.1111/jgs.70313)
Supplement: Supplementary file 1 — Data S1: Supporting Information. [file JGS-74-781-s001.pdf]

Online Supplementary Material

Assessing Older Adults' Adherence to Appropriate Polypharmacy: Selection of Outcome Measures for Intervention Trials

Table of Contents

Table S1. Summary of sources and search parameters for outcomes and outcome measurement instruments (OMIs) 2

Table S2. A list of journals from which editors were identified and older people's foundations, associations, organisations and charities contacted to enhance public participation 3

Supplement S1. The consensus meeting script for academics, healthcare professionals, journal editors, methodologists (i.e. experts) and public participants 6

Table S3. Reasons for selecting 'no' and 'uncertain' in the first round of the Delphi questionnaire 16

Table S4. Outcome measurement instruments suggested after the first round of the Delphi questionnaire 33

Table S5. Participants' silent reflection responses about each outcome measurement instrument resulting from the consensus meetings 38

Figure S1. A comprehensive flow chart summarising the identification and selection of OMIs for a COS for clinical trials targeting interventions to improve adherence to appropriate polypharmacy in older people. 42

Table S1. Summary of sources and search parameters for outcomes and outcome measurement instruments (OMIs)

Parameter	Details
**Primary source**	Cochrane systematic review: Cross *et al*. (2020), PRISMA-compliant
**Studies in Cochrane review**	50 publications (n=14,269 participants)
**Outcomes/OMIs from Cochrane review**	Adherence (48 studies: 28 subjective, 20 objective); HRQoL (14 studies); Healthcare utilisation (23 studies); Adverse events (6 studies); Cost-effectiveness (4 studies)
**Supplementary search purpose**	To identify studies published after Cochrane review search date
**Databases searched**	PubMed, Google Scholar
**Supplementary search period**	October 2018 – October 2021
**Search terms**	'adherence', 'polypharmacy', 'multiple medications', 'qualitative study', 'core outcome set', 'semi-structured interview', 'patient perspectives', 'focus group' (and combinations thereof)
**Studies included for OMI compilation**	Patton *et al.* (2021). OMIs identified: MARS-5, DPPR, MPR, EQ-5D-5L
**Studies excluded**	Bekker *et al.* (2021): No OMIs applied (qualitative study exploring patient perspectives on outcome domains)

DPPR: Daily Polypharmacy Possession Ratio; **EQ-5D-5L:** The 5-level EQ-5D questionnaire; **HRQoL:** Health-related quality of life; **MARS:** Medication Adherence Report Scale; **MPR:** The Medication Possession Ratio; **OMIs:** outcome measurement instruments; **PRISMA:** Preferred Reporting Items for Systematic reviews and Meta-Analyses;

- Bekker CL, Bossina S, de Vera MA*, et al.* Patient perspectives on outcome domains of medication adherence trials in inflammatory arthritis: an international OMERACT focus group study. *J Rheumatol*. 2021; **48**: 1480–1487.
- Cross AJ, Elliott RA, Petrie K, Kuruvilla L, George J. Interventions for improving medication-taking ability and adherence in older adults prescribed multiple medications. *Cochrane Database Syst Rev*. 2020; **5**: CD012419.
- Patton DE, Pearce CJ, Cartwright M*, et al.* A non-randomised pilot study of the Solutions for Medication Adherence Problems (S-MAP) intervention in community pharmacies to support older adults adhere to multiple medications. *Pilot Feasibility Stud*. 2021; **7**: 18.

Table S2. A list of journals from which editors were identified and older people's foundations, associations, organisations and charities contacted to enhance public participation

Journal editors:

A list of journals concerned with adherence, drug safety, patient education, polypharmacy, gerontology and ageing from which editors were identified:

1) Age and Ageing	17) Drug Safety	33) Journal of Aging Studies
2) Ageing & Society	18) Drugs & Aging	34) Journal of Anti-Aging Medicine
3) Ageing International	19) European Geriatric Medicine	35) Journal of Family Medicine and Primary Care
4) Ageing Research Reviews	20) European Journal of Ageing	36) Journal of Integrated Care
5) Aging and Disease	21) Experimental Aging Research	37) Journal of the American Geriatrics Society
6) Aging and Mental Health	22) Experimental Gerontology	38) Journal of Women & Aging
7) Aging Clinical and Experimental Research	23) Geriatric Nursing	39) Patient Education and Counselling
8) Aging Medicine and Healthcare	24) Geriatrics	40) Patient Preference and Adherence
9) Aging, Neuropsychology, and Cognition	25) GeroScience	41) Quality in Ageing and Older Adults
10) Aging-US	26) International Journal of Alzheimer's Disease	42) Research on Aging
11) BMC Geriatrics	27) International Journal of Integrated Care	43) The International Journal of Aging and Human Development
12) Canadian Geriatrics Journal	28) International Journal of Older People Nursing	44) The Journal of Nutrition, Health & Aging
13) Clinical Interventions in Aging	29) Journal of Aging & Social Policy	45) The Lancet Healthy Longevity
14) Clinics in Geriatric Medicine	30) Journal of Aging and Health	46) Therapeutic Advances in Drug Safety
15) Current Aging Science	31) Journal of Aging and Physical Activity	
16) Current Geriatrics Reports	32) Journal of Aging Research	

Public participants:

Older people’s foundations, associations, organisations and charities approached to enhance public participation in the consensus meeting:

The United Kingdom (UK):

1. Involve https://www.involve.org.uk/
2. The National Care Association https://nationalcareassociation.org.uk/
3. British Geriatrics Society https://www.bgs.org.uk/
4. Re-engage https://www.reengage.org.uk/
5. Engage with Age https://engagewithage.org.uk/
6. The University of the Third Age https://www.u3a.org.uk/
7. Volunteer Now NI https://www.volunteernow.co.uk/
8. AgeUK https://www.ageuk.org.uk/
9. Scottish Older People’s Assembly http://www.scotopa.org.uk/aboutus.asp
10. Rotary https://www.rotarygbi.org/
11. Carers UK https://www.carersuk.org/
12. Guts UK https://gutscharity.org.uk/advice-and-information/symptoms/heartburn-and-reflux/
13. British Heart Foundation https://www.bhf.org.uk/
14. Diabetes UK https://www.stroke.org.uk/finding-support
15. Mind https://www.mind.org.uk/
16. Versus Arthritis https://versusarthritis.org/
17. Stroke Association https://www.stroke.org.uk/finding-support
18. Cancer Research UK https://www.cancerresearchuk.org/
19. Be Part of Research https://bepartofresearch.nihr.ac.uk/taking-part/uk-research-registries/
20. Women’s Institute https://www.thewi.org.uk/
21. Cochrane consumer network https://consumers.cochrane.org/

Australia:

1. Consumers Health Forum https://chf.org.au/
2. National Seniors Australia https://nationalseniors.com.au/
3. The Heart Foundation https://www.heartfoundation.org.au/

The United States:

1. International Association for Public Participation https://www.iap2usa.org/
2. National Alliance for Caregiving https://www.caregiving.org/about/

Canada:

1. Carers Canada https://www.carerscanada.ca/
2. Age-well https://agewell-nce.ca/

3. The Canadian Association on Gerontology https://cagacg.ca/
4. Canadian Longitudinal Study on Aging (CLSA) https://www.clsa-elcv.ca/

Ireland:

1. The Irish Platform for Patient Organisations, Science and Industry https://www.ipposi.ie/
2. Friends of the Elderly https://friendsoftheelderly.ie/
3. Age Action Ireland https://www.ageaction.ie/
4. Care Alliance Ireland https://www.carealliance.ie/index
5. Age Action https://www.ageaction.ie/
6. Family Carers Ireland https://familycarers.ie/carer-supports/carer-support-groups
7. Diabetes Ireland https://www.diabetes.ie/
8. Irish Heart Foundation https://irishheart.ie/

Europe:

1. Eurocarers https://eurocarers.org/
2. European Institute for Public Participation https://participedia.net/organization/201#
3. European Lung Foundation https://europeanlung.org/en/
4. EUPATI https://eupati.eu/

Supplement S1. The consensus meeting script for academics, healthcare professionals, journal editors, methodologists (i.e. experts) and public participants

The consensus meeting script for experts

1. Pre-elicitation or introduction stage

Good morning/afternoon. My name is Hanadi, and I'm a PhD research student from the School of Pharmacy, Queen's University Belfast. I'll be moderating today's session. Thank you for taking the time to participate in this meeting. I greatly appreciate it. Our consensus meeting will last approximately one hour and 40 minutes.

As you are aware, this study is part of a larger project that hopes to identify a list of outcome measurement instruments to be used alongside a Core Outcome Set (COS) in future trials to evaluate interventions focusing on adherence to appropriate polypharmacy.

Three studies have been completed so far. In the first study, a group of outcomes was compiled from other research, which were then discussed with key stakeholders during a series of semi-structured interviews. The key stakeholders agreed that 13 outcomes seemed to be important. In the second study, these 13 outcomes were included in a Delphi consensus exercise aimed at reaching agreement on the most important outcomes for a COS. Academics, healthcare professionals, journal editors, and public members completed a series of three online questionnaires that contained information about these 13 outcomes. As a result, seven out of the 13 outcomes reached consensus from the Delphi study. In the third study, a Nominal Group Technique approach was used to discuss and refine the list of outcomes, which has resulted in a list included six outcomes, namely 1) medication adherence across multiple medications, 2) treatment burden, 3) health-related quality of life, 4) all adverse events or side effects, 5) healthcare utilisation, and 6) cost-effectiveness. In the present study, we are interested in reaching consensus on measurement instruments or methods used to assess outcomes in the context of adherence to appropriate polypharmacy to be used with the COS. Outcome measurement tools or instruments are used to quantitatively or qualitatively measure a patient's health status change in clinical practice. Outcome measurement instruments could be subjective or objective measures. Objective measurement instruments are standardised, unbiased and quantifiable methods of administration and scoring. They are recorded using physical examinations and diagnostic instruments to identify specific diseases, conditions or outcomes (e.g. laboratory measures, X-rays, machine or electronic monitoring devices) where no personal judgment is involved. Subjective instruments, on the other hand, depend mainly on personal judgement and consist mainly of Patient Reported Outcome Measures (PROMs). PROMs are questionnaires completed by the patients and are used to collect health-related details like satisfaction with the treatment, treatment load, signs, symptoms, and quality of life. Patients complete these questionnaires without any input or change from healthcare professionals, caregivers, friends or family members. However, sometimes, proxies, such as healthcare professionals, caregivers, friends or family members, can complete these questionnaires on the patient's behalf if the patient is unable to do so themselves for some health reasons. An example of a

PROM is the Medication Adherence Report Scale (MARS), used to evaluate the degree to which individuals follow their prescribed medications for different health conditions. However, the problem lies in the large number of these PROMs and outcome measurement instruments or methods, making it difficult to choose the most accurate and suitable instrument/method. Therefore, we conducted a Delphi exercise study before this meeting, which some of you might have participated in, to vote on the two highest-scoring PROMs for the following outcomes: 1) medication adherence across multiple medications, 2) treatment burden, and 3) health-related quality of life, as well as the two highest-scoring adherence objective measures. In this meeting, you will vote for one measurement instrument or method that you think is the most suitable for measuring outcomes included in this COS.

Before we kick off, I'd like to emphasise the importance of open conversation. Please feel free to express your opinions and thoughts, ask any questions or provide comments; this will really help our discussion. Please be aware that despite interacting with each other during the meeting, all participants' identities won't be disclosed when outcome measurement instruments/methods are voted on and the final consensus is reached. Everything discussed here is confidential; therefore, no participants' identities should be revealed outside this meeting. In our discussion today, it's important to note that there are no wrong or right responses, all perspectives are valued, and all points of view are important to us.

To give you a general idea about today's meeting, I'll briefly describe how this meeting will run. It will consist of three stages. The first stage is the **silent reflection of ideas**, which is done individually, whereby each participant will take time to reflect on each outcome measurement instrument and method and write down their responses. The second stage is the **group discussion stage**. In this stage, you'll share your perspectives about each measurement instrument. You'll also discuss and compare your views about each instrument by considering the views expressed by other participants. Lastly, in the final **voting stage**, you'll vote on whether you think each measurement instrument should be used to measure each outcome by choosing 'yes' or 'no'.

I. Silent generation of ideas

To start off, I'll send an email, containing a link to a workbook embedded in an online system called Sogolytics®. In this workbook, all the identified measurement instruments/methods related to each outcome, along with their information cards, will be presented one after the other. *Information cards* contain descriptive information about each outcome measurement instrument, such as abbreviations or alternate names, description (or definition), area assessed, recall period, scoring information, completion time, costs, and required equipment. They also contain all the essential details, such as the methods, formulas, advantages, disadvantages or instruments used to assess some outcomes (e.g. electronic devices used to measure adherence or methods/approaches used to evaluate healthcare utilisation and all adverse events and side effects). You'll be asked to select one outcome measurement instrument for each outcome based on your experience, knowledge, background, perspective, and the provided information. In addition, for outcomes that do not have PROMs (or questionnaires) to evaluate them or are measured by other methods, you will be provided with a suggestion on how to measure them. In the workbook, you'll be asked whether you

agree with the provided suggestion or if you have an alternative perspective/suggestion. You'll be given 50 minutes to reflect upon all measurement instruments. Once you click on 'submit' in the silent reflection workbook, a report of your notes will instantly appear on the 'thank you' page to help you recall your responses and share them with other fellow participants. Now, let's kick off with the first stage. I'd also be grateful if you could all mute your microphones at this time.

[*Send the silent reflection workbook to participants*].

[*Ensure that all participants received their workbooks (e.g. Have you all received your workbooks?*)].

[*Give participants time to complete their responses and answer any questions*].

Thank you very much for taking the time to complete the workbook and submitting your responses. Now, you'll be given a 10-minute break before the next group discussion stage.

[*Give participants a ten-minute break*]

3. <u>Group discussion</u>

Welcome back! Thank you once again for your valuable input and for completing the workbooks. Your silent reflection notes results are now opened in front of you. I would invite any of you who might be willing to explain and share your views on each instrument or measurement method. Please listen to other participants' experiences and perspectives about each outcome. You might find it helpful to make notes during this stage if you would like to do so.

Now, let's start with outcome Y, along with its measurement instruments.

- Would any of you be willing to share your perspective about outcome Y, and its measurement instrument *1*?

[*Wait for any participant who is willing to share their perspectives*].

- [*Participant 1*], thank you for your willingness to share your perspectives. Let's start with your response. Could you please let us know what you think about measurement instrument *1* and whether it should be used to measure outcome *Y*?

[*Give Participant 1 time to respond and share his/her views*].

- Would any of you like to comment on measurement instrument/method *1*?

[*Wait for another participant who is willing to share their perspectives*].

- [*Participant 2*], thank you very much. Could you please let us know what you think about measurement instrument *1*?

[*Continue until no perspectives remain*].

[*Continue in the same manner for each outcome until all measurement instruments/methods/PROMs are discussed*].

- Would any of you like to comment on what's been raised on any of the measurement instruments or methods?

[*Wait for participants' responses*].

Follow-up questions (used <u>if</u> needed).

- Would you like to respond to any points raised by any of the participants?
- Would you like to clarify your own responses further?
- Does anyone have any additional comments?

[*Wait for participants' responses*].

Thank you all for your valuable input and for sharing your views about each instrument in the list.

4. <u>Voting</u>

Thank you, everyone, for your comments on all instruments. Now, let's move on to the last stage, the voting stage. I'll send on an email to each of you with your identification code and a link to the questionnaire platform containing the list of all outcomes and their measurement instruments to vote. Please click on the link and decide if each of the instruments for each outcome should be used to measure the outcome by indicating 'yes' or 'no'. Your voting will help us finalise the list of measurement instruments related to the COS that should be used in intervention studies aiming to improve adherence to appropriate polypharmacy.

Please note that if you select 'Yes', this means the instrument should be included to measure an outcome in the COS, whereas if you select 'No', this means that this instrument shouldn't be included in the COS. For an instrument to be included, consensus (agreement) will be achieved when it's been given a vote of 'Yes' by 80% or more of participants who've completed the questionnaire <u>AND</u> 'No' by less than 20% of those who've completed it. Similarly, if an instrument is given a vote of 'No' by 80% or more of the participants <u>AND</u> 'Yes' by 20% or less of the participants, it will be excluded from the final COS and will not be used to measure the outcome. If you have any questions, please don't hesitate to ask.

[*Send the voting questionnaire to participants*].

[*Wait for participants to vote on all instruments*].

6. <u>Closing the consensus meeting</u>

This brings us to the end of today’s meeting. Does anyone have any additional comments that you’d like to make? Or any final questions?

[*Wait for participants’ responses and answer any questions*].

<u>Consensus meeting 1</u>

Before we wrap up, I’d like to let you know that another meeting such as this will be held. Once the second meeting is concluded, I’ll send you a link containing a report with the final list of outcomes, along with the instruments that all participants agreed upon in the two meetings.

Once again, many thanks for your participation in this study.

<u>Consensus meeting 2</u>

Before we wrap up, I’d like to let you know that based on the results from this meeting and an earlier meeting which took place on [Insert day], I’ll send you a link containing a report with the final list of outcomes, along with the instruments that all participants have agreed in the two meetings.

Once again, many thanks for your participation in this study.

Table of Contents

The consensus meeting script for public members

1. Pre-elicitation or introduction stage

Good morning/afternoon. My name is Hanadi, and I'm a PhD research student from the School of Pharmacy, Queen's University Belfast. I'll be in charge of today's session. Thank you for taking the time to participate in this meeting. I greatly appreciate it. Our consensus meeting should last approximately one hour and 40 minutes.

As you are aware, this study is part of a project that hopes to produce a list of outcome measurement instruments to measure the effect of a treatment, action or method (sometimes called interventions) on an outcome. In order to see if this new treatment or method (or, in other words, again, intervention) really works, healthcare professionals use *outcome measurement instruments* or methods to measure the effect of the intervention on outcomes. The *outcome* is the effect or result of using a treatment or receiving medical care (i.e. intervention) to help people aged 65 years and older take their multiple medicines (≥4 regular medicines) as directed. *Outcome measurement instruments* measure any change in a patient's health, medication-taking or behaviours to confirm whether the treatment has given positive results, such as better control of blood pressure, negative outcomes (e.g. severe nausea) or no noticeable change. Researchers classified measurement instruments as objective and subjective measures. *Objective measurement instruments* are unbiased instruments that are not influenced by people's perspectives and judgments. These objective instruments use numbers and devices to identify specific diseases, conditions or outcomes. Such outcome measurement instruments could be devices that record if patients open the bottle and take the medication, laboratory measures, like blood pressure (120/80), or numbers and percentages, such as the number of times a patient is admitted to the emergency department. Subjective measurement instruments measure what patients feel and say about their health, concerns, why they stop taking the medicines, or why they visit the healthcare facility. Subjective measurement instruments, such as a single question or a questionnaire, are called *Patient-Reported Outcome Measures (PROMs)*. PROMS are questionnaires containing a list of questions completed by patients. Examples are questionnaires to measure the quality of life, satisfaction with the healthcare provided, medication-taking behaviours, signs, symptoms, and emotions. An example of a PROM is the Medication Adherence Report Scale (MARS), used to measure the extent to which individuals take their prescribed medications for different health conditions.

Does anyone have any questions or comments about the terms?

[*Wait for participants' responses*].

Three studies have been completed so far to agree on six outcomes that can be used in the *COS (Core Outcome Set)*. A *COS* is the minimum number of outcomes that should be used and measured in a specific health area, so that all researchers use the same outcomes in the same types of studies. The list of outcomes included in the COS was: 1) medication adherence across multiple medications, 2) treatment burden, 3) health-related quality of life, 4) all adverse events or side effects, 5) healthcare utilisation, and 6) cost-effectiveness. The

definition of these outcomes is presented under each of them in the workbooks and the voting questionnaires that you'll receive during today's meeting. In this current study, we are interested in identifying which measurement instruments, PROMs, or methods should be used to measure these outcomes in trials to assess if a new intervention which aims to improve medication-taking (or adherence) to multiple medicines works. Before this meeting, a group of participants voted on measurement instruments. In today's meeting, you'll give your thoughts and vote for one outcome measurement instrument considered the most suitable for measuring each outcome in the COS.

Before we kick off, I'd like to emphasise the importance of open conversation. Please feel free to express your opinions and thoughts, ask any questions or provide comments; this will really help our discussion. Please be aware that despite interacting with each other during the meeting, all participants' identities won't be disclosed when outcome measurement instruments are voted on and the final agreement is reached. Everything discussed here is confidential; therefore, no participants' identities should be revealed outside this meeting. In our discussion today, it's important to note that there are no wrong or right responses, all perspectives are valued, and all points of view are important to us.

To give you a general idea about today's meeting, I'll briefly describe how this meeting will run. It will consist of four stages. The first stage is the **silent reflection of ideas**, which is done individually, whereby each participant will take time to reflect on each outcome measurement instrument and write down their responses. The second stage is the **group discussion stage**. In this stage, you'll share your perspectives about each measurement instrument. You'll also discuss and compare your views about each instrument. Lastly, in the final **voting stage**, you'll vote on whether you think each measurement instrument should be used to measure each outcome by choosing 'yes' or 'no'.

I. Silent generation of ideas

To start off, I'll send an email containing a link to a workbook that is part of an online system called Sogolytics®. In this workbook, all the measurement instruments and methods related to each outcome, along with their information cards, will be presented in turn. *Information cards* contain information about each measurement instruments, such as abbreviations or alternate names (if available), recall period (the time over which people are asked to remember information from the past, such as last week or last month), how long it takes to complete, costs, equipment needed to use it and other details on the instrument to make it clearer to you. You'll be asked to choose one outcome measurement instrument for each outcome according to your views and the provided information. *Feasibility* means how easy and user-friendly it is to use a specific outcome measurement instrument in the setting where the research is taking place, considering factors like time, necessary equipment, costs, and how quick and simple it is to use. For outcomes that do not have PROMs (or questionnaires) to measure them, or if they are measured in other ways or approaches, you will be provided with a suggestion on how to measure them. In the workbook, you will be asked whether you agree with the provided suggestion or if you have an alternative perspective/suggestion. You'll be given 50 minutes to think about all measurement instruments. Please submit your notes by clicking on 'submit' when you've finished. Once you click on 'submit' in the silent

reflection workbook, a report of your notes will instantly appear on the ‘thank you’ page to help you remember your responses and share them with other participants. Now, let’s kick off with the first stage. I’d also be grateful if you could all mute your microphones at this time.

[*Send the silent reflection workbook to participants*].

[*Ensure that all participants received their workbooks (e.g. Have you all received your workbooks?*)].

[*Give participants time to complete their responses and answer any questions*].

Thank you very much for taking the time to complete the workbook and submitting your responses. You’ll be given a 10-minute break before the next group discussion stage.

[*Give participants a ten-minute break*]

3. <u>Group discussion</u>

Welcome back! Thank you once again for your valuable input and for completing the workbooks. Your notes’ results are now opened in front of you. I welcome any of you who might be willing to share your views on each instrument or measurement method. Please listen to other participants’ experiences and perspectives about each outcome. You might find it helpful to make notes during this stage if you would like to do so.

Now, let’s start with outcome Y, along with its measurement instruments.

- Would any of you be willing to share your perspective about outcome Y, and its measurement instrument *1*?

[*Wait for any participant who is willing to share their perspectives*].

- [*Participant 1*], thank you for your willingness to share your perspectives. Let’s start with your response. Could you please let us know what you think about measurement instrument *1* and whether it should be used to measure outcome *Y*?

[*Give Participant 1 time to respond and share his/her views*].

- Would any of you like to comment on measurement instrument/method *1*?

[*Wait for another participant who is willing to share their perspectives*].

- [*Participant 2*], thank you very much. Could you please let us know what you think about measurement instrument *1*?

[*Continue until no perspectives remain*].

[*Continue in the same manner for each outcome until all measurement instruments/methods/PROMs are discussed*].

- Would any of you like to comment on what's been raised on any of the measurement instruments or methods?

[*Wait for participants' responses*].

Follow-up questions (used <u>if</u> needed).

- Would you like to respond to any points raised by any of the participants?
- Would you like to clarify your own responses further?
- Does anyone have any additional comments?

[*Wait for participants' responses*].

Thank you all for your valuable input and for sharing your views about each instrument in the list.

4. <u>Voting</u>

Thank you, everyone, for your comments on all instruments. Now, let's move on to the last stage, the voting stage. I'll send on an email to each of you with your identification code and a link to the questionnaire containing the list of all outcomes and their measurement instruments to vote. Please click on the link and decide if each of the instruments or methods for each outcome should be used to measure the outcome by indicating 'yes' or 'no'. Your voting will help us finalise the list of measurement instruments related to the COS that should be used in intervention studies aiming to improve adherence to appropriate polypharmacy.

Please note that if you choose 'Yes', this means the instrument should be included to measure an outcome in the COS, whereas if you choose 'No', this means that this instrument shouldn't be included in the COS. For an instrument to be included, it needs to achieve a vote of 'Yes' by 80% or more of participants who've completed the questionnaire <u>AND</u> 'No' by less than 20% of those who've completed it. Similarly, if an instrument is given a vote of 'No' by 80% or more of the participants <u>AND</u> 'Yes' by 20% or less of the participants, it will be excluded from the final COS and will not be used to measure the outcome. If you have any questions, please don't hesitate to ask.

[*Send the voting questionnaire to participants*].

[*Wait for participants to vote on all instruments*].

6. <u>Closing the consensus meeting</u>

This brings us to the end of today's meeting. Does anyone have any additional comments that you'd like to make? Or any final questions?

[*Wait for participants' responses and answer any questions*].

<u>Consensus meeting 1</u>

Before we wrap up, I'd like to let you know that another meeting such as this will be held. Once the second meeting is concluded, I'll send you a link containing a report with the final list of outcomes, along with the instruments that all participants agreed upon in the two meetings.

Once again, many thanks for your participation in this study.

<u>Consensus meeting 2</u>

Before we wrap up, I'd like to let you know that based on the results from this meeting and an earlier meeting which took place on [Insert day], I'll send you a link containing a report with the final list of outcomes, along with their instruments that all participants have agreed in the two meetings.

Once again, many thanks for your participation in this study.

Table S3. Reasons for selecting 'no' and 'uncertain' in the first round of the Delphi questionnaire

Note: All comments have been reported exactly as they were presented on the SoGolytics® platform, without any modifications, and there has been no attempt to correct spelling, grammar, or punctuation. A summary has been added for each instrument.

Could you please provide your reasons for selecting 'No' or 'Uncertain'? (optional).

Outcome #1: Medication adherence across multiple medications

1- The Brief Medication Questionnaire

One of the concerns regarding the BMQ is the lack of a timeline, and that it is too descriptive. Participants stated that they were not familiar with it. Several commented that it was quite long, potentially too complex a time-consuming, and burdensome to fill out for people on more than six medicines or for those over 65 taking multiple medications. Others said it would be difficult for patients with more than 15 different drugs and that people's beliefs about medication could vary for each one, making the BMQ unfeasible factor. Some participants noted that it required detailed and accurate medicines reconciliation and questioned whether a carer could complete it. The recall period was seen as too short or not specified. Many said that the BMQ focused on medication knowledge or management rather than adherence, did not measure the construct of adherence, and that there was no way to sum the score across a regimen. A few participants questioned its practicality given patients' knowledge about medicines and scoring time, while others said it was only easy to complete for people taking one medication. Some were uncertain about its evidence base or contribution to the Delphi topic.

	**Reasons**
1	*"A major concern with this is the lack of timeline. How many days did you take it. Too descriptive"*
2	*"I am unsure of its validity"*
3	*"Can you imagine doing this "brief" questionnaire for patients with 15 - 20 different drugs???"*
4	*"Quite long. #2-#3 could of the BMQ could potentially be removed."*
5	*"Unsure of the sensitivity of this measure"*
6	*"For people >65 taking multiple medications may be difficult to complete, overestimating non-adherence"*
7	*"items pertain to issues with medication management, which may not always directly lead to non-adhere [sic]"*
8	*"not familiar enough with it"*
9	*"Full completion is burdensome for people on >6 medicines, use of section 1 is helpful + 2a & 3c"*

10	*"People's beliefs about medication can vary for each medication, therefore the BMQ is unfeasible"*
11	*"The BMQ seems more likely to question medication knowledge and problems with medication management"*
12	*"Requires detailed and accurate medicines reconciliation, not clear if carer can complete"*
13	*"Recall in one week is to [sic] short for chronic disease."*
14	*"potentially too complex and time consuming"*
15	*"What is it?"*
16	*"The patients' knowledge about their medications and scoring time can limit the practicality."*
17	*"It is a good scale but it does not measure the construct of adherence"*
18	*"it asks about each medicine individually but there's no way about summing the score across the regim [sic]"*
19	*"Problem with all these questionnaires: only easy to fill out for people taking one medication"*
20	*"I don't know what the current evidence indicates re its contribution to the topic of this Delphi"*
21	*"recall period not specified"*
22	*"A major concern with this is the lack of timeline. How many days did you take it. Too descriptive"*

2- The Medication Adherence Report Scale (MARS)

Participants believed that the always–never scale choices were subjective, with no specified recall period. It was also considered very time-consuming for every medication, too limited (e.g. no information regarding the initiation phase of adherence), required payment, too vague and unclear concerning the extent of non-adherence. They were also unsure regarding the sensitivity of the MARS questionnaire.

	**Reasons**
1	*"The always never scale is so subjective. My always can be someone's rarely. And just few reasons"*
2	*"Again: very time consuming for every med, but better than BMQ"*
3	*"Too vague, not clear on extent of nonadherence"*
4	*"Unsure of the sensitivity of this measure"*
5	*"It is not free of charge. I think that instruments publicly available should be prioritized."*
6	*"Multiple versions of the MARS may be needed for each medication"*
7	*"Not comprehensive, e.g., you don't get information about the initiation phase of adherence."*
8	*"Not clear if carer can complete, MARS10 needs modified as example for asthma not widely applicable"*

9	*"The internal consistency is low."*
10	*"Not an outcome - process measure - limited validity. Adherence is a 'predictor' of clinical outcome"*
11	*"Self-report has poor correlation with actual adherence. There are better options with better reliabi [sic]"*
12	*"it asks about each medicine individually but there's no way about summing the score across the regim [sic]"*
13	*"I don't know what the current evidence indicates re its contribution to the topic of this Delphi"*
14	*"recall period not specified; answering scale is not specific (what is often?);"*
15	*"The always never scale is so subjective. My always can be someone's rarely. And just few reasons"*

3- The Medical Outcome Study (MOS) – Specific Adherence Scale

Participants believed the MOS Adherence Scale was lengthy, vague, and too burdensome and time-consuming to complete for multiple medications. It was more suitable for certain conditions, such as cardiovascular diseases and diabetes, making it unsuitable for other chronic conditions. Participants noted that this questionnaire encompassed items reflecting general adherence behaviour rather than asking about multiple medications. Others reported that the recall period was long and that they were unfamiliar with it.

	**Reasons**
1	*"This is not specific to medications. This is treatment regime including patient appointments"*
2	*"length and mainly pertains to CVD, diabetes"*
3	*"It is too long, make it difficult to be completed by participants"*
4	*"too long"*
5	*"Not sure the lifestyle part is needed if we talkt [sic] about drug adherence?"*
6	*"Not specific to adherence to medication"*
7	*"Quite vague, could see patients interpreting not doing exactly what the doctor said as too broad"*
8	*"Too specific for certain chronic conditions"*
9	*"It is not specific of medication and therefore it is too time consuming."*
10	*"not specific to medecations [sic]"*
11	*"Better adherence scales available"*
12	*"focus is (a) to broad [sic] in terms of adherence, (b) too narrow in terms of conditions"*
13	*"It is a disease specific scale"*
14	*"The items reflects general adherence behaviour not specific to multiple medications."*
15	*"not familiar enough with it"*
16	*"Full use is lengthy/burdensome, many questions are not medication adherence specific"*
17	*"Multiple versions of the MOS may be needed for each medication"*
18	*"Not as frequently used or known about"*

19	*"Scale does not focus on medicines"*
20	*"Self reporting for older patients should be simple."*
21	*"too disease specific"*
22	*"Adherence is not an outcome - it is an input and process measure"*
23	*"Relatively long, and measures specific recommendations and behaviours (limited to 3 conditions).."*
24	*"Self report has poor correlation with observed adherence. Also there are better options."*
25	*"It asks across the regimen but it's about doing as you're told"*
26	*"I don't know what the current evidence indicates re its contribution to the topic of this Delphi"*
27	*"quiet [sic] long; long recall period"*
28	*"This is not specific to medications. This is treatment regime including patient appointments"*

4- The Morisky Medication Adherence Scale (MMAS)

Participants believed the MMAS was vague, subjective, not comprehensive, and expensive to purchase, with a cumbersome licensing strategy that hindered its use by researchers with limited funding. They were also unsure about the use of this questionnaire across multiple drugs. It had little evidence of validity and reliability, therefore the key validation paper had been retracted.

	**Reasons**
1	*"Not sure whether enough information is covered by the questionnaire"*
2	*"This one is too subjective and just 4 reasons for non-adherence. Classic example - cost?"*
3	*"It is not free limiting its use to researchers with limited funding"*
4	*"The Moriskey scale is not open access - this should not be supported in todays research world."*
5	*"Challenges with obtaining this scale. Socially desirable responses likely"*
6	*"Cost associated with use of this scale, copyright issues"*
7	*"Too vague"*
8	*"Major issues in approval and licensing"*
9	*"It does not perform well. Has little evidence of validity and reliability."*
10	*"Not open access and high cost to purchase"*
11	*"licensing fee may obstruct use and lead to issues for evidence synthesis in future"*
12	*"my understanding is that the developer of this scale makes it difficult for researchers to use this"*
13	*"Very generic categorical responses, very strict, which may overestimate the lack of adherence. Compl"*
14	*"The key validation paper has been retracted. https://onlinelibrary.wiley.com/doi/10.1111/jch.14718"*
15	*"Not comprehensive enough, e.g., no information on inititiation phase."*

16	*"Multiple meds may be taken for a health condition, integrity of authors, cost"*
17	*"Copyright problems"*
18	*"It is simple scale, but the author was not easy to permit the use!"*
19	*"unclear how to implement across multiple drugs. cost is substantial"*
20	*"This cannot be used as has now been withdrawn. Journal rescinded the original paper recently"*
21	*"Costs of use"*
22	*"Legal issues around the use of this scale is a major barrier."*
23	*"Dubious quality. Cost."*
24	*"Harsh licence strategy with high costs (or penalties), not what scientific collaboration should be"*
25	*"I don't know what the current evidence indicates re its contribution to the topic of this Delphi"*
26	*"no option any more since the publ. was retracted in 2023 (https://retractionwatch.com)"*

5- Pill counts

Participants believed that pill counting was complex, unfeasible, inaccurate, and too burdensome and time-consuming to complete for multiple medications and large populations. It was also reported that pill counting did not confirm actual adherence or medication-taking and could alter behaviour if the patient was aware that counting may be undertaken. It does not allow for understanding the actual causes of non-adherence.

	**Reasons**
1	*"Too time consuming. Also, people may just dump the pills before coming to pharmacy"*
2	*"complexity in patients with ++ medications"*
3	*"Pill counts do not confirm actual adherence and can change behaviour (if patient aware)"*
4	*"It's just a surrogate - did the patient really apply the drug?"*
5	*"time consuming and difficult to implement in large-scale population studies"*
6	*"Too combersome [sic] with large numbers of medications and not sensitive enough."*
7	*"Feasibility concerns"*
8	*"Issues of validity and reliability"*
9	*"It is not fully reliable, it is time consuming and not always feasible."*
10	*"methodological issues (e.g. applying arbitrary cut-off values across several medicines)"*
11	*"Its a crude measure of adherence, results are inaccurate, does not capture all types of medicines"*
12	*"Costly method that may also fail to detect if the patient is taking the wrong regimen"*
13	*"This may be an unfeasible measure for multiple medications"*
14	*"only formulation specific"*

15	*"So time consuming + participant need to retains pack but potentially give most accurate account"*
16	*"does not allow understanding the causes of non-adherence"*
17	*"Process measure"*
18	*"Due to its disadvantages"*
19	*"Less valid compared to EMDs, but sometimes more feasible (finances)"*
20	*"I don't know what the current evidence indicates re its contribution to the topic of this Delphi"*
21	*"with the given disadvantages"*

6- Electronic Monitoring Devices (EMDs)

Participants believed that EMD administration was impractical and complex to use, especially for older patients taking multiple medications. It was noted that patients might need to use more than one device to record each medicine, which was also considered prohibitive. EMDs did not necessarily reflect actual medication-taking, as patients might just open the device without taking the medicine.

	**Reasons**
1	*"Patients need to be in a position to use the device. Who fixes the device to medicines container?"*
2	*"Too expensive and intrusive. Also, people may just open the box without taking the medicine"*
3	*"May not be available to all patients"*
4	*"complexity and feasibility with older adults taking ++ medications"*
5	*"They are expensive and can change behaviour"*
6	*"Might be beneficial for trials, but expensive and complicated"*
7	*"Not easy to measure multidrug adherence and too expensive"*
8	*"Not feasible with high numbers of medication usage"*
9	*"Feasibility issues"*
10	*"Perhaps for RCTs of efficacy but may be less practical in determining effectiveness"*
11	*"It is not fully reliable, it is costly and not always feasible."*
12	*"not suitable for all meds"*
13	*"unsuitable for polypharmacy; tech complexity - exclusion; ethics - surveillance (nb swallow format)"*
14	*"challenging to use in polypharm, are intervention themselves so cannot capture real life adherence"*
15	*"Concerns about feasibility in setting of cognitive syndromes."*
16	*"Not applicable in patients with polypharmacy, expensive and difficult to manage in the elderly."*
17	*"This may be an unfeasible measure for multiple medications"*
18	*"technology can fail"*
19	*"Change of meds packaging may impact on existing patient established systems to support adherence"*

20	*"to [sic] expensive"*
21	*"wholly impractical in a polypharmacy context"*
22	*"Process measure"*
23	*"Their costs and disadvantages outweigh their benefits."*

Retrospective electronic databases

Despite being feasible and cost-effective in real-world settings, retrospective electronic databases were deemed inaccurate indirect measures reliant on the accuracy of prescription pharmacy records, which led to overestimation or underestimation of medication adherence. Participants believed these tools failed to reflect actual medication-taking, as filling a prescription did not confirm that the medicine was taken. It was reported that retrospective electronic databases did not capture the reasons for non-adherence and were time-consuming to calculate for multiple medications and large populations.

A few participants stated that MPR did not account for overlapping periods or gaps between refills, while others indicated that DPPR was more suitable than MPR for measuring polypharmacy.

7- Medication Possession Ratio (MPR) – Retrospective electronic databases

	**Reasons**
1	*"may be too crude / non-specific in trial setting"*
2	*"Again: did the patient really take the meds, but low effort in the pharmacy"*
3	*"Could be used as an adjunct measurement"*
4	*"not a direct measure"*
5	*"In ideal world - yes but in reality database records are often spotty and inaccurate"*
6	*"DPPR when available is more suitable for polypharmacy"*
7	*"It may over or understimate adherence"*
8	*"DPPR preferable to MPR with approp. database, overlap included but only issues/access captured"*
9	*"Lack of information on whether the patient is following the guideline adequately. Complementary tool"*
10	*"Possible overestimation of adherence"*
11	*"Useful if dispensing data widely available, prescribing data unreliable due to repeat dispensing"*
12	*"does not allow understanding the causes of non-adherence"*
13	*"Process measure"*
14	*"Time consuming to calculate for each medication"*
15	*"Indirect measurement, but very feasible and cost-effective"*
16	*"I don't know what the current evidence indicates re its contribution to the topic of this Delphi"*
17	*"with the given disadvantages"*

8- Proportion of Days Covered (PDC) – Retrospective electronic databases

	Reasons
1	*"may over estimate adherence"*
2	*"may be too crude in trial setting"*
3	*"Should better be done at the point of contact, the pharmacy, too retrospective"*
4	*"Can be used as an adjunct"*
5	*"not a direct measure"*
6	*"In ideal world - yes but in reality database records are often spotty and inaccurate"*
7	*"DPPR when available is more suitable for polypharmacy"*
8	*"DPPR preferable as uses standardised paramters for calculation & a continuous index of adherence"*
9	*"Lack of information on whether the patient is following the guideline adequately. Complementary tool"*
10	*"Like AdhereR, need to consider meds supplied by multiple places e.g. post-admission supply"*
11	*"does not allow understanding the causes of non-adherence"*
12	*"Process measure"*
13	*"Time consuming to calculate for each medication"*
14	*"See MPR"*
15	*"I don't know what the current evidence indicates re its contribution to the topic of this Delphi"*
16	*"with all challenges: it is a valuable approach if you have a large population you can include"*

9- Daily Polypharmacy Possession Ratio (DPPR) – Retrospective electronic databases

	Reasons
1	*"Is this a covert method? Will such a method be approved by ethics committee?"*
2	*"may be too crude in trial setting"*
3	*"Only accurate if the same pharmacy is used - and used at all."*
4	*"Can be used as an adjunct"*
5	*"not a direct measure"*
6	*"In ideal world - yes but in reality database records are often spotty and inaccurate"*
7	*"Lack of information on whether the patient is following the guideline adequately. Complementary tool"*
8	*"Not certain how this measure is derived."*
9	*"Like AdhereR, need to consider meds supplied by multiple places e.g. post-admission supply"*
10	*"does not allow understanding the causes of non-adherence"*
11	*"Time consuming to calculate for each medication"*
12	*"see MPR"*

13	*"I am not familiar with this measure and cannot comment re how it compares with other measures"*

Outcome #2: Treatment burden

1- Treatment Burden Questionnaire (TBQ)

Participants believed the TBQ was complex for older people to complete due to the terminology or language used and was not freely available, with a licensing process that hindered its use by researchers with limited funding. Participants were also aware of the sensitivity issues.

	**Reasons**
1	*"It is not free limiting its use to researchers with funds"*
2	*"Not directly measuring adherence"*
3	*"Good content, but 15 questions are a lot..."*
4	*"Unsure of the sensitivity of this measure"*
5	*"Long and complex questionnaire. Not free."*
6	*"user fee may cause issues for evidence synthesis in future"*
7	*"not familiar enough with it"*
8	*"Can be complex for older people to complete due to terminology/language used"*
9	*"not medicine specific"*
10	*"Unsure how sensitive to change items will be when considering multiple medicines"*
11	*"130 items for the olders?"*
12	*"I don't know what the current evidence indicates re its contribution to the topic of this Delphi"*
13	*"answering scale not optimal (10: too many options + not labelled => different interpretations)"*
14	*"Sensitivity issues and it's not freely available"*

2- Multimorbidity Treatment Burden Questionnaire (MTBQ)

Participants noted that the MTBQ scope was too broad and did not directly measure adherence or multiple medications. Others stated that they were unfamiliar with this questionnaire and were uncertain about its sensitivity when measuring multiple medications.

	**Reasons**
1	*"Not directly measuring adherence"*
2	*"Better to have the focus on medication. Scope too wide"*
3	*"not familiar enough with it"*
4	*"not medicine specific"*
5	*"Unsure how sensitive to change items will be when considering multiple medicines"*
6	*"Not detailed enough to do anything with"*
7	*"Can't see the questions?"*
8	*"I don't know what the current evidence indicates re its contribution to the topic of this Delphi"*

3- Patient Experience with Treatment and Self-management Questionnaire (PETS)

Participants expressed several concerns regarding the PETS questionnaire. The majority considered it complex, lengthy, and burdensome to complete, making it unfeasible for older people. Others believed its focus or scope was too broad and that it did not specifically measure multiple medications.

	**Reasons**
1	*"quite complex, some questions are not directly related to the measurement of adherence"*
2	*"Too long?"*
3	*"too many questions"*
4	*"It is too long"*
5	*"Not directly measuring adherence"*
6	*"YES to the short version!"*
7	*"Too many items"*
8	*"Length of instrument may be a challenge"*
9	*"too long, feasibility concerns"*
10	*"too long"*
11	*"Better to have the focus on medication. Scope too wide"*
12	*"Long and burdensome questionnaire."*
13	*"conditions around user fee are not clear"*
14	*"May not feasible, longer administration time"*
15	*"not familiar enough with it"*
16	*"lengthy/burdensome to complete"*
17	*"Potentially overly laborious."*
18	*"I am not familiar with this measure"*
19	*"lengthy"*
20	*"Too long"*
21	*"To [sic] many questions for the elderly!"*
22	*"quite burdensome on respondents"*
23	*"Many items"*
24	*"I don't know what the current evidence indicates re its contribution to the topic of this Delphi"*

4- The Living with Medicines Questionnaire-3 (LMQ-3)

As with the PETS questionnaire, the LMQ-3 was also deemed generic, complex, lengthy, and burdensome to complete, making it unfeasible for older people. Others stated that they were unfamiliar with this tool or that it required the author's permission to use, which would limit its use in research. However, a few participants indicated that this questionnaire was more specifically related to polypharmacy, as it focused more on medication burden.

	**Reasons**
1	*"Too long?"*

2	*"too many questions"*
3	*"It is too long"*
4	*"I like the score approach, but too extensive"*
5	*"Too many items"*
6	*"Length of instrument may be a challenge"*
7	*"too long, feasibility concerns"*
8	*"last section is likely not relevant, upper section seems useful"*
9	*"Unsure of the sensitivity of this measure"*
10	*"Long and burdensome questionnaire."*
11	*"aspects of treat. burden besides medicines are not captured (if treat. burden is defined as above)"*
12	*"May not feasible, longer administration timem [sic]"*
13	*"not familiar enough with it"*
14	*"lengthy/burdensome to complete"*
15	*"I am not familiar with this measure"*
16	*"less familiar with this tool. More generic instrument"*
17	*"Very long but questions more relevant to medicines than PETS"*
18	*"The availability is limited by the author."*
19	*"quite burdensome on respondents"*
20	*"Many items"*
21	*"I don’t know what the current evidence indicates re its contribution to the topic of this Delphi"*

Outcome #3: Health-related quality of life

The EQ-5D questionnaires

Despite being a series of brief questionnaires, the EQ-5D was considered general and irrelevant to measuring the influence of multiple medications on adherence. A few participants were unfamiliar with the questionnaires and reported that they were insensitive to change. The EQ-5D-5L was preferable to the EQ-5D-3L, as it included more comprehensive items with better sensitivity.

1- The 3-level EQ-5D questionnaire (EQ-5D-3L)

	Reasons
1	*"this questionnaire is intended for other purposes and is not specific to measurement of adherence"*
2	*"long process to obtain it"*
3	*"No specific enough"*
4	*"Good tool - but do we need this for adherence measurement?"*
5	*"Unsure of the sensitivity"*
6	*"The 5L version is available."*
7	*"less sensetive [sic] to detect changes than EQ-5D-5L"*
8	*"not familiar enough with it"*
9	*"not sensitive to medication-related changes in quality of life, needed for calculation of QALYs"*
10	*"The 5-level EQ-5D questionnaire (EQ-5D-5L) is similar and more complete."*
11	*"The questions are too general and do not consider the influence of medication."*
12	*"Good brief measure, general QoL not necessarily meds related"*
13	*"5D-5L version preferable"*
14	*"Less sensitivity and 5L"*
15	*"the SF-36 seems to be the industry standard consistent with other work"*

2- The 5-level EQ-5D questionnaire (EQ-5D-5L)

	Reasons
1	*"this is a generic questionnaire, not aimed at measurement of adherence"*
2	*"Personally prefer the 3-item version above for efficient screening"*
3	*"There are a number of generic HRQoL measures. Really need to know more about sensitivity"*
4	*"not familiar enough with it"*
5	*"As above"*
6	*"uncertain of the value of the broader range of responses compared to 3 Level version"*
7	*"The questions are too general and do not consider the influence of medication."*
8	*"Extract options not required"*
9	*"not sensitive to change"*

The Short Form questionnaires

The Short-Form (SF) questionnaires were deemed generic, non-specific, and irrelevant to measuring the influence of multiple medications on adherence. A few participants were unfamiliar with these questionnaires, while others reported that they were insensitive to change, lengthy compared with the EQ-5D questionnaires, and expensive to purchase, with a cumbersome licensing process hampering their use by researchers with limited funding. The SF-36 was considered very lengthy, with multiple questions and a scale ranging from 0 to 100, making it cumbersome and complex to complete for older people.

3- The Short Form-12 (SF-12)

	**Reasons**
1	*"this is a generic, non-specific questionnaire"*
2	*"cost to use"*
3	*"The scale from 0 - 100 seems difficult to me for older patients"*
4	*"I'm not certain of the psychometric properties of SF12 vs 36"*
5	*"There are a number of generic HRQoL measures. Really need to know more about sensitivity"*
6	*"Longer than EQ-5D, not free to use and utility tariffs unavailable for many countries."*
7	*"license fee may skew usage opportunities and cause issues for evidence synthesis in future"*
8	*"not familiar enough with it"*
9	*"The Short Form-36 (SF-36) is similar and more complete."*
10	*"Not sure this tool is used as often as EQ-5D"*
11	*"The questions are too general and do not consider the influence of medication."*
12	*"Multiple questions potentially not be applicable to study population"*
13	*"not sensitive to change"*
14	*"might be overly long for intended population"*
15	*"if I remember correctly: the total score can only be calculated for pts without missing data."*

4- The Short Form-36 (SF-36)

	**Reasons**
1	*"generic and non-specific"*
2	*"Length of questionnaire"*
3	*"too involved"*
4	*"Far too extensive - and not centered upon adherence itself"*
5	*"Too many items"*
6	*"? length and feasibility"*
7	*"There are a number of generic HRQoL measures. Really need to know more about sensitivity"*
8	*"Too long, not free to use and utility tariffs unavailable for many countries."*
9	*"Lenght [sic] - burdensome for participants (especially when SF-12 is available)"*

10	*"not familiar enough with it"*
11	*"lengthy to complete"*
12	*"Not sure this tool is used as often as EQ-5D and if SF used probably SF-12 preferred"*
13	*"Good measure of QoL but MRB-QoL captures both so less burdensome on participants"*
14	*"To [sic] long"*
15	*"too burdensome"*
16	*"Overly complex, thereby adding burden to patients or research team"*
17	*"might be overly long for intended population"*

5- The Medication-Related Burden Quality of Life (MRB-QoL) questionnaire

Participants expressed some concerns regarding the MRB-QoL questionnaire. The majority considered it complex, lengthy, and burdensome to complete, particularly for older people. However, others believed it covered two outcomes: treatment burden and health-related quality of life, making it more appropriate for evaluating non-adherence caused by administering many medications (i.e. medication burden). Some participants stated that they were unfamiliar with this questionnaire and were unsure about its sensitivity.

	**Reasons**
1	*"Too long?"*
3	*"too many items"*
4	*"Length of questionnaire"*
5	*"too involved"*
6	*"Too many items and this scale is not widely used in previous studies"*
7	*"similar to treatment burden"*
8	*"There are a number of generic HRQoL measures. Really need to know more about sensitivity"*
10	*"Long instrument. Covered by "Treatment burden" and QoL instruments."*
11	*"concept of med. related burden QoL seems to measure something different to QoL as defined above"*
12	*"Relevant to medication burden but newer and need more psychometric evidence."*
13	*"not familiar enough with it"*
14	*"I think it is more appropriate to evaluate medication burden"*
15	*"I am not familiar with this measure"*
16	*"Not familiar with this instrument for QoL"*
17	*"mixed outcomes measured"*
18	*"to [sic] long"*

Outcome #4: All adverse events and side effects

The number of undesired consequences of the intervention (i.e. adverse events or side effects) that result from administering multiple medications in older patients

Although measuring all adverse events and side effects was deemed important, participants stated that patients could not distinguish whether the side effect was caused by the medication or by the disease itself, making it unfeasible to collect for multiple medications.

	Reasons
1	*"Patients will find difficulty to attribute adverse effects to specific treatment"*
2	*"many can not self-identify ADEs, or do not attribute them to medications"*
3	*"It may be difficult to patients to rate relatedness. It should be easy to report."*
4	*"This may be unfeasible to collect for multiple medications & patients may have difficulty reporting"*
5	*"Not sure if intervention being referred to here is the adherence intervention or the medicines"*
6	*"unclear how to be formulated"*

Outcome #5: Healthcare utilisation

The number or percentage (name of the utilised service) during/in (time period) that result from administering multiple medications in older patients

Participants believed that *"the number or percentage (name of the utilised service) during/in (time period) that result from administering multiple medications in older patients"* was undefined and ambiguous due to the availability of different services. It also failed to distinguish between healthcare utilisation resulting from medication administration and/or other causes. Filling a prescription for stable patients did not always indicate that these patients utilised healthcare services or systems.

	Reasons
1	*"this tool is general and does not distinguish between utilisation due to medicines or other reasons"*
2	*"stable patients may use more refill request without actually utilizing the service"*
3	*"not specific"*
4	*"Not a sensitive measure in those who are multimorbid and/or frail. Many factors influence this"*
5	*"Somewhat unclear description, makes it seem like population level"*
6	*"How are these to be determined?"*
7	*"the approach appears slightly too undefined/unsystematic, depending on the context, data source, etc"*
8	*"difficult to measure / and to assess true causality"*
9	*"suspect will be difficult in many cases to link utilisation specifically to meds"*

10	*"how operationalised?"*
11	*"The above statement does not make sense. Healthcare utilisation needs to be measured"*
12	*"I assume that this is relevant but it needs to specified which services are relevant"*

Outcome #6: Cost-effectiveness

Consulting a health economist about the most appropriate method

Participants believed that measuring cost-effectiveness could be a barrier for research, as the concept was not properly defined. Another participant questioned how it would be operationalised, while a few considered *"consulting a health economist about the most appropriate method"* unsuitable because economic outcomes were not confined to cost-effectiveness alone.

	**Reasons**
1	*"The main perspective for the evaluation and the interested stakeholders will affect the perspective"*
2	*"I think it is not properly defined. Efficiency is another outcome. We did not discuss effectiveness."*
3	*"Not always available, so ideal but may be a barrier to research"*
4	*"Operationalisation?"*
5	*"Q does not make sense. Economic outcomes important - not solely cost-effectiveness"*

Table S4. Outcome measurement instruments suggested after the first round of the Delphi questionnaire

Note: All comments have been reported exactly as they were presented on the SoGolytics® platform, without any modifications, and there has been no attempt to correct spelling, grammar, or punctuation.

Outcome #1: Medication adherence across multiple medications

Please feel free to provide suggestions for another outcome measurement instrument to measure 'medication adherence across multiple medications', considering the context of use (adherence to appropriate polypharmacy) and the target population (older people over 65). (optional)

	**Suggestions**	**Reason for exclusion**
1	*"simple to use use [sic] by patients with disabilities e.g. dexterity, eyesight, health literacy, reading"*	Not relevant
2	*"The Adherence to Refills and Medications Scale"*	1 (n=7)
3	*"Medication Adherence Reasons Scale"*	1 (n=4)
4	*"Even if it's a research environment - the instruments are too time consuming. We need targeted tools"*	Statement
5	*"For MPR, you could calculate MPR for each of the medications to determine extent of adherence"*	Statement
6	*"The Adherence to Refills and Medications Scale"*	2
7	*"Medication Adherence Universal Questionnaire, disease specific [sic] measures"*	1 (n=4)
8	*"I would also measure initiation and early discontinuation."*	Statement
9	*"I suggest Medication Adherence Universal Questionnaire (please see https://mauq.org/)"*	2
10	*"The adherence to refills and medications scale (ARMS)"*	3
11	*"Voils"*	Suggested by one participant only
12	*"As beliefs & medication taking can vary for each med, then med specific [sic] versions may be needed"*	Not relevant/Only general measures
13	*"See MAR-Scale and ARMS"*	2, 4
14	*"Multiple discretized MPR/PDC was calculated using a dichotomized adherence rate (≥80% yes/no)"*	Statement
15	*"The Medication Adherence Reasons Scale (MAR-Scale)"*	3
16	*"the adherence to refills and medications scale"*	5
17	*"Medication Adherence Universal Questionnaire (MAUQ) https://doi.org/10.1007/s11096-023-01612-x"*	3 (n=4)
18	*"Check MAR-Scale https://www.ncbi.nlm.nih.gov/pmc/articles/PMC6612984/"*	4
19	*"It is not useful to perform intervention [sic] in practice."*	Statement

20	*"The Adherence to Refills and Medications Scale. https://www.sciencedirect.com/science/article/pii/S1"*	6
21	*"Krousel-Wood Medication Adherence Scale DOI: 10.1097/HJH.0000000000001955"*	Suggested by one participant only
22	*"-"*	–
23	*"Maybe Medication Adherence Universal Questionnaire"*	4
24	*"It wld [sic] be useful to have evidence summary re [sic] the above tools & [sic] their performance re [sic] this population/"*	Statement
25	*"ARMS"*	7

Outcome #2: Treatment burden

Please feel free to provide any suggestions for another outcome measurement instrument to measure 'treatment burden', considering the context of use (adherence to appropriate polypharmacy) and the target population (older people aged more than 65). (optional)

	Suggestions	Reason for exclusion
1	*"keep it simple"*	Statement
2	*"I would also need to know which tool is validated to make a decision!"*	Statement
3	*"Burden of the caregiver (if any) should be taken into account as well."*	Statement
4	*"Simple pill count - how many pills does the person have to take every day"*	Statement
5	*"I"*	
6	*"LMQ is generic , medicine specific [sic] , widely validated"*	Statement
7	*"MRB-QOL https://doi.org/10.1136/bmjopen-2017-018880"*	"Repetitive/ already exist in health-related quality of life
8	*"All of the tools so far are not outcomes. They are measures of process and predictors"*	Statement
9	*"SIMS"*	Suggested by one participant only
10	*"Same comment as before - summary of evidence of these tools would be useful to inform decision"*	Statement

Outcome #3: Health-related quality of life

Please feel free to provide any suggestions for another outcome measurement instrument to measure 'health-related quality of life', considering the context of use (adherence to

appropriate polypharmacy) and the target population (older people aged more than 65). (optional)

	Suggestions	Reason for exclusion
1	*"questionnaire should focus on adherence to medicines and should be aimed for elderly to be specific"*	Statement
2	*"I prefer a tool that has medication at its center if we talk about med adherence"*	Statement
3	*"See above. If all equally sensitive then would opt for the shortest"*	Statement
4	*"Could consider the ICECAP-O (although is [sic] is broader 'wellness' rather than HRQoL)"*	Suggested by one participant only

Outcome #4: All adverse events and side effects

The number of undesired consequences of the intervention (i.e. adverse events or side effects) that result from administering multiple medications in older patients

Please feel free to provide suggestions for another outcome measurement instrument/method (if available) to measure 'all adverse events and side effects', considering the context of use (adherence to appropriate polypharmacy) and the target population (older people aged more than 65). (optional)

	Suggestions	Reason for exclusion
1	*"May consider measuring medication-related harm arising from ADR, medication errors, and nonadherence"*	Statement
2	*"consideration of the severity of the adverse event should be included e.g. for ADRs-mild, mod, sever [sic]"*	Statement
3	*"Why no scale here? How are these to be determined?"*	Statement
4	*"Agree these should be measured but it might be additional burden to complete this information."*	Statement
5	*"NCI PRO-CTCAE developed for cancer patients, but potentially useful in adapted version"*	Disease-specific measure/Suggested by one participant only/Only general measures

Outcome #5: Healthcare utilisation

The number or percentage (name of the utilised service) during/in (time period) that result from administering multiple medications in older patients

Please feel free to provide any suggestions for another outcome measurement instrument to measure 'healthcare utilisation', considering the context of use (adherence to appropriate polypharmacy) and the target population (older people aged more than 65). (optional)

	Suggestions	Reason for exclusion
1	*"Ideally use specific measures related to medicines. Utilisation could be due to the condition"*	Disease-specific measure/Only general measures
2	*"For GP visits important to differentiate type of visit e.g. prescription only, GP or nurse visit etc"*	Statement
3	*"Why no scale here?"*	Statement
4	*"Number of rehospitalizations"*	Statement
5	*"Could consider a more holistic measure such as healthy days at home (see PMID 31708403)"*	Suggested by one participant only
6	*"FIMA"*	Suggested by one participant only

Outcome #6: Cost-effectiveness

Consulting a health economist about the most appropriate method

Please feel free to provide suggestions for another outcome measurement instrument/method (if available) to measure 'cost-effectiveness', considering the context of use (adherence to appropriate polypharmacy) and the target population (older people aged more than 65). (optional)

	Suggestions	Reason for exclusion
1	*"Define the perspective to be used. Prioritise the items to be considered as inputs and outputs."*	Statement
2	*"Provided there is consultation and appropriate model is chosen"*	Statement
3	*"How to be determined?"*	Statement
4	*"Not properly defined. CE compares the relative efficiency (cost per health gain) of 2 interventions."*	Statement
5	*"very difficult to differentiate between some of the assessment instruments across several outcomes"*	Statement

6	*"Why is this question relevant to this survey?"*	Statement
7	*"-"*	Empty
8	*"The semantics of some of these questions are problematic. The use of brackets is unhelpful"*	Statement

Table S5. Participants' silent reflection responses about each outcome measurement instrument resulting from the consensus meetings

Note: All comments have been reported exactly as they were presented on the SoGolytics® platform, without any modifications, and there has been no attempt to correct spelling, grammar, or punctuation.

Participant No.	Silent reflection responses
**Medication adherence across multiple medications**	
**Subjective adherence measure: ARMS**	
**Experts**	
P1	*"It is simple and subjective method. Of course the question is what is exactly meausered* [sic]*? visit to pharmacy?"*
P2	*"Yes, I consider it relevant to include a patient reported adherence assessment. I agree that the ARMS can provide reliable data."*
P3	*"yes* [sic] *but might need some adjustments. I think, a timeframe could be usefull* [sic] *because most patients are not able to remember a longer period of time. Additionally, all Likert-scale* [sic] *should have the same ranking. Also, it contains a lot of questions."*
P4	*"Yes. I like ARMS. Good for low literacy. Covers a range of points (thorough). Does require the client to remember if they have ever missed doses etc.; therefore, may pick up 'some' non-adherence."*
P5	*"The scale is not clear, what is the definition of "most" and "some"? More clarification is needed, eg. once a week? once* [sic] *a month etc. Is the questionnaire being used face to face or via telephone? It is common in elderly patients to have hearing and cognitive impairment. Not sure if you want to record the type of medicines that patients tend to miss or adjust themselves. The questionaire* [sic] *does not explore why patients are missing their medication.*<br>*I feel that this questionnaire can be used to monitor or to screen for suitable patients, but adjustment is needed."*
**Public participant**	
PP1	*"Yes, though I don't care for the word 'Careless'* [sic] *though accurate it may feel like blame. 'Forget' or 'not get around to' might be more forgiving questions."*
**Objective adherence measures: DPPR**	

Participant No.	Silent reflection responses
**Experts**	
P1	*"Could be used. But we must be aware that the result is actually showing the situation in "home drug depot"*
P2	*"Yes, apart from PROM assessment, I'm very much in favour to add a more objective assessment. As a data source, I would make a plea for claims data instead of pharmacy prescription data, which are nog* [sic] *linked between pharmacies in all countries."*
P3	*"Hm, not clear. It might be usefull* [sic] *to have this information and excludes* [sic] *desired answers. But it depends very much on the information in the pharmacy and accurate dosing information from the presciber* [sic].
P4	*"No. I don't believe DPPR should be used. Just because a client is re-ordering medications doesn't mean they are actually adherent to medications."*
P5	*"I think that this is not an accurate way of measuring or monitoring whether the patients are taking medicines. Some pharmacies ordered medications on behalf of the patients."*
**Public participant**	
PP1	*"No, not much use, no confirmation on medication taking, what's collected from the chemist could or* [sic] *sold or given to another."*
**Treatment burden**	
**MTBQ and the LMQ-3**	
**Experts**	
P1	*"The interaction among the patients and healthcare prof. is* [sic] *always reccomendable* [sic]. *Of course we should be trained to avoid some personal misunderstanding. The relationship should be built-"*
P2	*"I would advice* [sic] *MTBQ for two reasons: 1. it is short 2. availability of validated versions in multiple languages"*
P3	*"Preference for the MTBQ. It is shorter for patients, easier to recieve* [sic] *the information wanted and translated so it can be used in several countries and comparision* [sic] *of results is much easier."*
P4	*"I believe MTBQ fit* [sic] *better with assessing treatment burden. It is also shorter for clients to complete compared to LMQ-3. Both are quite similar."*
P5	*"I feel the multiple choice answers for MTBQ are very subjective. What does "a little difficult" and "Quite difficult" mean? Vague. I feel the options for LMQ-3*

Participant No.	Silent reflection responses
	is [sic] *more suitable. Unfortunately, I was unable to see an example of the LMQ-3, but based on the option, I prefer LMQ-3."*
**Public participant**	
PP1	*"I would go with MTBQ. I am not allowed to answer the questions of the questionnaires I gave in details* [sic]. *What is the point in worrying about the question/questionnaire when there is no concern for the answer? I might be able to adhere to the regime one day because the home help arrived on time to give me water to take my medication with or the person administering the injection arrived on time. another* [sic] *day there might be a technical difficulty that prevented me correctly following the regime, like I was on the bus to the hospital appointment at the set time..... Allow someone free text to answer if you have any interest in getting accurate information. Questionnaires generally are not good, we need to explain to the chemist"*
**Health-related quality of life**	
**MRB-QoL and EQ-5D-5L**	
**Experts**	
P1	*"In a real life* [sic] *the simple is better! Of course that more items gives you more data. The MRB-QoL is better for research to know simple differences in adherence to polypharmacy."*
P2	*"For reasons of comparability to previous studies, I would advice* [sic] *to use EQ-5D-5L. It can be used to measure cost-effectiveness. However, the MRB-QoL is more specific"*
P3	*"QoL ist* [sic] *always so very diffficult* [sic] *and often you cannot see any changes. My preference is the EQ-5D-%L* [sic] *because it is short, robust, and translated. It has already been used in many trials. MRB-Qol cannot be used if one quote* [sic] *is missing, that is always difficult when patients fill such formats."*
P4	*"I prefer MRB-QoL. It covers more. However, I do recognise that it would take longer to complete that* [sic] *the EQ-5D-5L and may put clients off, particularly those with low literacy levels."*
P5	*"I feel MRB-QOL has more questions, so it will give more information about the patient, but EQ-5D-5L is easier to complete. No preference. MRB-QOL is better for polypharmacy."*
**Public participant**	
PP1	*"MRB-QoL for medications"*

Participant No.	Silent reflection responses
**All adverse events and side effects**	
**The number of undesired consequences of the intervention (i.e. adverse events or side effects) that result from administering multiple medications in older patients**	
**Experts**	
P1	*"This is* [sic] *obligation for healthcare workers. We must look for them also to improve our advices* [sic] *to patient (I was doing it through my 40 years practice!)."*
P2	*"Yes. But, it would be helpful to develop a standardised measurement of the number and type of adverse events and side effects (compare PRO-CTCAE to assess (toxicologic) symptoms in cancer patients."*
P3	*"Would not use this because it is to* [sic] *diffcult* [sic] *for patients to connect side-effects to drugs most of the time. And it is not very specific e.g. if you have a patient reporting dizziness and it is because the RR [Respiratory Rate] is too low, maybe or it is only related to too low fluid intake? Very difficult and imprecise."*
P4	*"It would be good to know all adverse events and side effects. Clients however may be non-adherent for other reasons."*
P5	*"Yes, because side effects can affect medicine adherence."*
**Public participant**	
PP1	*"Yes, medicine list should be regularly monitored for a build up* [sic] *of various agents that might build up over time and cause unintended side effects."*
**Healthcare utilisation**	
**The number or percentage of a specified utilised service during/in a specified time period that results from administering multiple medications in older patients**	
**Experts**	
P1	*"Yes! This will discover one of important* [sic] *reason* [sic] *of adherence to polypragmasia [polypharmacy]!"*
P2	*"I agree that health care utilisation should be assessed, but again preferably in a standardised way. Recall period is essential here, with (to my knowledge) 6 months being the suitable time window. The German FIMA Questionnaire works quite well."*
P3	*"No, there is not always a clear relation between adherence and healthcare utilisation and utilisation is often very difficult do* [sic] *assess and it needs a clear and precise clarification of health-care services types."*

Participant No.	Silent reflection responses
P4	*"Knowing healthcare utilisation could be useful however, it may indicate adherence and it may not. it* [sic] *is more likely to indicate worsening of their condition."*
P5	*"No, I feel that this is not accurate because some patients may be undiagnosed for some conditions."*
**Public participant**	
PP1	*"Yes. Necessary."*
**Cost-effectiveness**	
**Consulting a health economist about the most appropriate method**	
**Experts**	
P1	*"Yes, but we should be aware not to use it for discriminatory reasons (to reject some expensive treatment because the patient is old!)."*
P2	*"This is really not my area of expertise."*
P3	*"No, cost-effectiveness is even more difficult to measure as health-care ulitisation* [sic] *and it is linked to it. And the most international health systems make this outcome very difficult to compare."*
P4	*"Cost-effectiveness could be useful, and is linked to outcome 5* [healthcare utilisation]*."*
P5	*"Not familiar with this, unable to comment."*
**Public participant**	
PP1	*"No, non-branded is ok* [sic] *if the branded is more expensive. not* [sic] *a relevant question when there is a cap on what we pay regardless of how expensive the medicine is."*

*Multiple professions are listed for some experts because they selected more than one job title.

ADRs: Adverse drug reactions; **CE:** Cost-effectiveness; **CEA:** Cost-effectiveness analysis; **COPD:** Chronic Obstructive Pulmonary Disease; **HF:** Heart failure; **HRQL:** health-related quality of life; **QOL:** Quality of life; **RCTs**: Randomised Controlled Trials; **SE:** Side effects.

PHASE 1: COMPILATION OF OUTCOME MEASUREMENT INSTRUMENTS

Sources Consulted
- Cochrane review (Cross *et al.,* 2020)
- Feasibility study (Patton *et al.,* 2021)
- COSMIN database systematic reviews (Møller *et al.,* 2020; Mendoza-Quispe *et al.,* 2023)

Initial Instruments Identified
Total: n=21 instruments

- **Thirteen PROMs:** BMQ, MARS, MOS-Specific Adherence Scale, MMAS, TBQ, MTBQ, PETS, LMQ-3, EQ-5D-3L, EQ-5D-5L, SF-12, SF-36, MRB-QoL.
- **Five Objective measures:** EMDs, Pill counts, MPR, PDC, DPPR.
- **Three Measurement methods:** For all adverse events and side effects, healthcare utilisation, cost-effectiveness.

PHASE 2: DELPHI CONSENSUS EXERCISE

Round 1 - Participant Recruitment
Invited: n=320 (academics, HCPs, methodologists, journal editors)

- Participated: **n=42 (13.4%)**
- Additional via snowball sampling: **n=2**
- Declined: n=26 (8.1%)
- No response: n=251 (78.4%)

Response rate: 93.3% (42/45 agreed participants)

Consensus In - Round 1
n=1 instrument

- All adverse events and side effects measurement method (85.7%).

Consensus out – After Round 1
n=5 instruments

- BMQ
- MOS-Specific Adherence Scale
- MMAS
- TBQ
- PETS

Reached 'consensus out' threshold (≥80% voted 'no')

Round 1 - New Instruments Suggested by Participants
n=3 PROMs added (suggested by ≥4 participants)

- ARMS (n=7 participants)
- MAUQ (n=4 participants)
- MAR-Scale (n=4 participants)

Round 2 - Instruments Evaluated
Total: n=19 instruments
- Fifteen carried forward from Round 1
- Three newly suggested instruments
- One already achieving consensus (adverse events)

Consensus In - Round 2
n=8 instruments
- ARMS (82.1%)
- MTBQ (94.9%)
- LMQ-3 (82.1%)
- EQ-5D-5L (82.1%)
- MRB-QoL (82.1%)
- Adverse events method (100%)
- Healthcare utilisation method (94.9%)
- Cost-effectiveness method (92.3%)

Added by Research Team
n=1 instrument
- DPPR (74.4%)

Included as most relevant objective adherence measure despite not reaching 80% threshold.

Consensus Out – After Round 2
n=10 instruments
- MARS, EMDs, Pill counts, MPR, PDC, MAUQ, MAR-Scale (adherence measures).
- EQ-5D-3L, SF-12, SF-36 (quality of life measures)

PHASE 3: CONSENSUS MEETING

Participant Recruitment
Invited: n=300 experts + 42 organisations/charities
- Experts agreed to participate: n=14 (4.6%)
- Additional via snowball (British Geriatrics Society): n=14
- Public participants: n=1 (via Irish Platform for Patient Organisations)
- Experts declined: n=41 (13.6%)
- No response: n=245 (81.6%)

Final Participants
n=6 total
- Experts: n=5 (academics, pharmacists, doctors)
- Public members: n=1

From 4 European countries: Croatia (n=1), Germany (n=2), Ireland (n=1), UK (n=2)

Instruments Discussed
n=9 instruments
Process: Silent reflection → Group discussion → Voting

**Final Consensus In**<br>**n=4 instruments**<br><br>(≥80% voted 'yes')<br>• ARMS (100%)<br>• MTBQ (100%)<br>• MRB-QoL (83.3%)<br>• Adverse events method (83.3%)	**Consensus Out – Excluded**<br>**n=5 instruments**<br><br>• DPPR (33.3%)<br>• LMQ-3 (33.3%)<br>• EQ-5D-5L (66.7%)<br>• Healthcare utilisation (50.0%)<br>• Cost-effectiveness method (33.3%)

FINAL CORE OUTCOME SET WITH MEASUREMENT INSTRUMENTS

1. **Medication adherence across multiple medications**
   - **Subjective:** Adherence to Refills and Medications Scale (ARMS)
   - **Objective:** No available measurement instrument
2. **Treatment burden**
   - Multimorbidity Treatment Burden Questionnaire (MTBQ)
3. **Health-related quality of life**
   - Medication-Related Burden Quality of Life questionnaire (MRB-QoL)
4. **All adverse events and side effects**
   - The number of undesired consequences of the intervention (i.e. adverse events or side effects) that result from administering multiple medications in older patients
5. **Healthcare utilisation**
   - No available measurement instrument
6. **Cost-effectiveness**
   - No available measurement instrument

ARMS: Adherence to Refills and Medications Scale; **BMQ:** Brief Medication Questionnaire; **DPPR:** Daily Polypharmacy Possession Ratio; **EMDs:** Electronic Monitoring Devices; **EQ-5D:** European Quality of Life 5-Dimension; **HCP:** Healthcare Professional; **LMQ-3:** Living with Medicines Questionnaire-3; **MAR-Scale:** Medication Adherence Reasons Scale; **MARS:** Medication Adherence Report Scale; **MAUQ:** Medication Adherence Universal Questionnaire; **MMAS:** Morisky Medication Adherence Scale; **MOS:** Medical Outcome Study; **MPR:** Medication Possession Ratio; **MRB-QoL:** Medication-Related Burden Quality of Life; **MTBQ:** Multimorbidity Treatment Burden Questionnaire; **PDC:** Proportion of Days Covered; **PETS:** Patient Experience with Treatment and Self-management; **PROM:** Patient-Reported Outcome Measure; **SF:** Short Form; **TBQ:** Treatment Burden Questionnaire.

Figure S1: A comprehensive flow chart summarising the identification and selection of OMIs for a COS for clinical trials targeting interventions to improve adherence to appropriate polypharmacy in older people.

See the linked version of this figure at: (https://adherence-to-appropriate-polypharmacy.github.io/Figure/).
